# How does practice modulate fake-production costs in a basketball task? Analyses of frequency distributions and mixture effects

**DOI:** 10.1007/s00426-025-02084-6

**Published:** 2025-03-06

**Authors:** Nils Tobias Böer, Christoph Schütz, Matthias Weigelt, Iris Güldenpenning

**Affiliations:** 1 Department of Sport & Health, Paderborn University, Warburger Str. 100, 33098 Paderborn, Germany; 2 Faculty of Psychology and Sports Science, Bielefeld University, Universitätsstraße 25, 33615 Bielefeld, Germany

## Abstract

The execution of incompatible actions imposes costs on action planning, commonly known as response-response incompatibility-costs. This phenomenon is also evident in sports: A basketball player who performs a pass in one direction whilst orienting the head into the contrary direction (pass with head fake) needs more time to initiate the action as if pass direction and head orientation are the same (pass without head fake).

The aim of this study was twofold: First, we present a re-analysis of the data from Böer et al. (Psychological Research 88:523–524, 2024) using mixture effect modelling (Miller, Behavior Research Methods 38:92–106, 2006) explore if fake-production costs manifest continuously (uniform effect) in all participants or if some participants show fake-production costs occasionally but substantially (mixed effect). Second, we collected data of a control group which was analysed with the previous data of the practice group and fitted initiation times (ITs) to an ex-Gaussian distribution.

The analysis of mixture effects revealed that most participants exhibited a uniform effect when they didn’t have time to mentally prepare the movement. This pattern was not changed by practice, suggesting fake-production costs can’t be overcome by practice alone without mental preparation time.

The analysis of mean ITs revealed improvements in the practice group but not in the control group, independent of the type of pass performed. The distribution analyses complemented these findings as it showed that the improvement in participants’ performance with increasing practice can mainly be attributed to a reduction of the exponential part of the distribution (parameter tau).

## Introduction

In many competitive sports athletes from different teams interact with each other in one-on-one duels. In such situations, deceptive actions can be used to gain an advantage over the opponent (Güldenpenning et al., 2017; Jackson & Cañal-Bruland, 2019). An example of a deceptive action is the head fake in basketball, where the attacking player turns the head into the direction opposite to the intended pass direction (Polzien et al., 2021). The head fake leads to slower responses and more response errors on the side of the observer, which is termed the head-fake effect (Kunde et al., 2011). Previous research on the head-fake effect focused on factors that modulate its size, for example, practice with the task (Güldenpenning, Schütz, et al., 2020b), cognitive load (Güldenpenning, Kunde, & Weigelt, 2020a), motor and visual training, as well as basketball expertise (Güldenpenning et al., 2022), and other factors (Friehs et al., 2019; Güldenpenning et al., 2024; Polzien et al., 2020, Weigelt et al., 2020; cf. Güldenpenning et al., 2017 for a review).

### Previous research on fake-production costs

In recent years, researchers began to investigate the cognitive costs associated with performing deceptive actions, the so-called fake-production costs (Böer et al., 2024; Güldenpenning et al., 2023; Kunde et al., 2019; Wood et al., 2017). Fake-production costs for the head fake in basketball are evident in increased initiation times (ITs) and higher error rates (ERs) when executing a pass with a head fake as compared to a pass without a head fake. These costs can be reduced when participants are given time to mentally prepare the movement with Interstimulus Intervals (ISIs) of 800 ms and more (Güldenpenning et al., 2023). Fake-production costs for the head fake are suggested to reflect response-response incompatibility-costs, as they are also known from bimanual actions (Hazeltine, 2005; Heuer, 1995; Peterson, 1965) and similarly have been shown to be reduced by mental preparation time (Heuer et al., 1998). Here, response-response incompatibility-costs occur, for example, when moving two fingers simultaneously, but one vertically and one horizontally (incompatible action), as compared to moving two fingers simultaneously in the same direction with the same trajectory (compatible action, cf. Hazeltine et al., 2003). The head fake also requires the execution of two movements which are spatially incompatible, namely a head turn to one side (e.g., to the left) and a passing movement to the other side (e.g., to the right).

Until now, fake-production costs have been largely disregarded in sport psychology research (for some exceptions, see Böer et al., 2024; Güldenpenning et al., 2023; Kunde et al., 2019; Wood et al., 2017), even though they are of great relevance for sport practice. Specifically, the costs when performing a head fake can determine its benefit, as an increase in the initiation time of the attacking player by 100 ms has been shown to increase the chance that the opponent would anticipate a deceptive movement by 10% (Kunde et al., 2019). Accordingly, it seems worthwhile investigating how and why fake-production costs can be decreased.

One factor that can reduce fake-production costs is extensive practice (i.e., five consecutive days of practice). In a recent study, we showed that novice participants, who elicited fake-production costs when given no (ISI 0 ms) or only little time (ISI 400 ms) to mentally prepare performing the passes with head fakes stabilized their performance [reduction of the variation coefficient of initiation times (IT_cv_)], especially from the first day of practice to the second day of practice, and reduced fake-production costs (Böer et al., 2024). Moreover, the study revealed that practice with the task led to a general improvement in ITs, independent of the type of pass, but also showed a greater improvement in ITs for passes with compared to passes without head fakes, as indicated by a reduction of the fake-production costs from day 1 to day 5 (see Fig. 5, p. 22). While participants were even able to eliminate these costs when given time to mentally prepare the movement (for an ISI of 400 ms), we only found a non-significant, descriptive reduction of fake-production costs through practice when participants had no mental preparation time (ISI 0 ms). Taken together, results suggested different effects of practice on the fake-production costs for the ISIs 0 ms and 400 ms. These effects of practice are most pronounced from day 1 to day 2, which was reflected by the steep learning curve well known for motor tasks (Dayan & Cohen, 2011; Hong et al., 2019). Thus, one day of practice substantially improves the ITs in passes with and without head fakes, however, as the learning curve flattens, significantly more practice might be necessary (i.e., over a longer period than 5 days) to achieve further visible increases in performance. However, it might also be that smaller effects of practice during day 2 and day 5 differ between individuals and/or are not visible in the mean value of the group. We therefore re-analysed the individual data of the participants of the study mentioned above (Böer et al., 2024) regarding so-called mixture effects (Miller, 2006), focusing on the ISIs 0 ms and 400 ms, where we found significant fake-production costs on the first day of practice.

### Analysis of mixture effects

The likelihood ratio test of mixture effects (Miller, 2006) is an analysis of the individual IT distributions. With this analysis at an individual level, we explore whether the fake-production costs from each participant stem from a relatively uniform slowing of all ITs when producing passes with head fakes compared to passes without head fakes (uniform effect), or from a combination of a minority of significantly slowed responses and a majority of equivalent ones (mixture effect). A uniform effect would suggest that response-response incompatibility-costs occur in most, if not all, trials (e.g., Compton & Logan, 1991; Osman et al., 2000). Conversely, a mixture effect would suggest that participants are capable of successfully executing passes with head fakes in most trials, and only sporadically exhibit the fake-production costs, but when they do, the extent is considerably larger. The analysis could help us understand if the reduction of fake-production costs through practice shown in our previous work (cf. Böer et al., 2024) differs between participants. The analysis may also uncover how cognitive processing evolves over the course of practice, for example, from a uniform effect where participants elicit fake-production costs in all trials to a mixed effect, where participants are able to overcome the fake-production costs in some trials.

### Control group

Moreover, since the original study could not clarify whether the general improvement of participants’ ITs, and especially the reductions in fake-production costs at the two short ISIs (0 ms, 400 ms) were based on the effects of practicing the passes or attributable to test–retest effects, we collected data post-hoc from a control group. The control group performed exactly the same experiment (i.e., participants performed 320 trials of passes with and without head fakes with randomized ISIs of 0 ms, 400 ms, 800 ms, 1200 ms), but were only tested on day 1 and day 5 without practicing in between. The data from both groups was analysed with the between-subjects factor *group *(control group, practice group) and the within-subjects factors *type of pass* (pass without head fake, pass with head fake), *ISI* (0 ms, 400 ms, 800 ms, 1200 ms), and *day* (day 1, day 5). If the reductions seen in the mean ITs of participants in the practice group are (at least partly) connected to practice with the task, the control group should display less improvements between day 1 and day 5, reflecting test–retest effects. Therefore, we expected significantly greater reductions of ITs in the practice group compared to the control group from day 1 to day 5, both for passes with and without head fakes. Also, we expected a greater reduction of the fake-production costs in the practice group at the ISIs 0 ms and 400 ms compared to the control group. The comparison between the practice and control groups was further analysed through repeated measures ANOVAs and distribution analyses, as described below.

### Analysis of initiation time distributions

﻿The analysis of the distribution of the initiation times was performed with the data of both groups, to further disentangle differences between them. Typically, reaction times (RTs)/ITs are not normally distributed but can be fitted to an ex-Gaussian distribution (Whelan, 2008), which is a mixture of a normal distribution and an exponential distribution. While most studies analyse mean RTs, this only reflects a precise value for the central tendency in the case of normally distributed distributions. However, RTs in experimental studies are often not normally distributed but also consist of an exponential part (Hockley, 1984; Hohle, 1965; Osmon et al., 2018; for more details see Luce, 1986). Therefore, an analysis of the whole RT distribution can yield valuable information about differences between conditions that may be overlooked in analyses focused solely on mean RTs.

The ex-Gaussian distribution can be described by the parameters mu and sigma, the mean and standard deviation of the Gaussian part, and the parameter tau, which represents the right tail of the distribution. The analysis of the ex-Gaussian parameters provides a succinct method to determine how the experimental manipulation impacts participants’ ITs. Specifically, this distribution analysis reveals whether the experimental manipulation shifts the IT distribution (i.e., a change in mu and/or sigma), elongates the tail of the distribution (i.e., a change in tau), or both. It has been proposed that the parameters of the ex-Gaussian distribution reflect cognitive processes, with some authors suggesting that the Gaussian part of the distribution (mu and sigma) encapsulate the aggregate of perceptual and motor processes (Kane & Engle, 2003; Luce, 1986; Mewhort et al., 1992). Tau, the exponential part of the distribution, has been discussed to reflect lapses of attention in some trials as evident in especially slow responses (i.e., the right tail of the distribution) and has been shown to be higher for cognitively demanding tasks (Heathcote et al., 1991; Hervey et al., 2006; Vasquez et al., 2018). However, some researchers have critically noted that it can be misleading to assume the parameters reflect specific cognitive processes and one has to be careful not to overinterpret them (Matzke & Wagenmakers, 2009; Osmon et al., 2018). But, when treated with some caution, the analysis of RT distributions can expand the knowledge generated by simple analysis of mean RTs and even uncover differences between experimental condition which are not visible in the analysis of mean values (Ratcliff, 1979).

Two key aspects of the ex-Gaussian parameters are relevant to this study:

1. In which parameter(s) of the ex-Gaussian distribution are the fake-production costs visible? As fake-production costs seem to be caused by response-response incompatibility (cf. Böer et al., 2024), we expect higher values in mu and sigma for passes with compared to passes without head fakes, reflecting higher demands on perceptual and motor processes (Luce, 1986; Mewhort et al., 1992).These differences in mu and sigma should be modulated by the duration of the ISIs, as we only found significant fake-production costs at the ISI 0 ms and 400 ms on the first day of practice (cf. Böer et al., 2024). We also expect a larger tau for passes with head fakes than for passes without head fakes. The higher cognitive demands for programming response-response incompatible movements could cause attentional lapses in some trials, which would be reflected in especially slow responses, prolonging the right tail of the distribution.
2. Are practice-based improvements in ITs found in our previous study (Böer et al., 2024), independently of the *type of pass*, due to a reduction of the Gaussian distribution parameter (distribution mean (mu) or spread (sigma)) or a reduction of long ITs, as seen in the exponential part of the distribution (distribution skew (tau))? Generally, repeated practice should lead to a familiarization of the task, which means that the cognitive resources required for the task decrease with increasing practice (Logan, 1992). Therefore, participants’ responses should show less variance and more homogeneity. Also, potential lapses of attention and extremely slow responses, which can be caused by a high task demand, should be reduced.

Accordingly, the following predictions were made for the distribution analyses: Both parameters of the distribution variation (spread (sigma), skew (tau)) should reduce from day 1 to day 5 for both groups, reflecting a general familiarization with the task. We also expect greater reductions of sigma and tau for the practice group than for the control group, reflecting practice-related performance improvements exceeding simple test–retest effects. As we previously argued, we believe performing head fakes is connected to higher cognitive demands and should therefore be reflected in higher values of tau for passes with compared to passes without head fakes. We expect no modulation of mu by the interaction of the factor *day* and the factor *group*, as we believe the reduction of ITs with increasing practice reported for the practice group in Böer et al. (2024) reflects mainly a stabilization of performance through decreasing variance (i.e., familiarization) and a reduction of the number of trials with extremely slow responses.

The analysis of mixture effects (Miller, 2006) performed on the data of the practice group should complement this analysis of the IT distribution parameters by allowing insight into individual differences between participants. A uniform effect, where participants consistently elicit fake-production costs, should be visible in greater values of the distribution mean (mu). Conversely, a mixture effect, where participants only show fake-production costs in some trials, should be visible in greater values of the distribution skew (tau).

## Methods

This study is mainly based on the data already reported in Böer et al. (2024). For a deeper understanding of the individual change of fake-production costs during the course of practice, we analysed the individual distributions of the mean initiation times (IT) with Millers MixTest (2006) (Data analyses part I and Results part I). Further, we present a comparison of the previous IT data with additionally post-hoc collected data of a control group, both as part of a variance analysis of the mean values of ITs and with a further distribution analysis (i.e., ex-Gauss analyses; data analyses part II and results part II). Before a detailed description of the different data analyses and its’ results are reported, the methodology of the experimental setup is outlined.

### Participants

In the practice group twenty-five sport science students from Paderborn University were tested on five consecutive days, but one participant’s data was excluded from further analysis due to missing data from the first measurement. The remaining twenty-four volunteers (5 females, mean age = 24.6 years, *SD* = 2.4) participated in the experiment for course credit.

To address a reviewer’s request for a control group, an additional group of thirty-two sport science students (14 females, mean age = 21.5 years, *SD* = 1.9) were tested on day 1 and day 5 of practice with a break of three waiting control days in between. Data collection and experimental setup were identical for both groups, the only difference was the number of days participants practiced. None of the participants of both groups had basketball experience beyond leisure sports activities. All participants reported normal or corrected-to-normal vision and all of them were naive to the expected outcome of this experiment. The study adhered to ethical guidelines from the German Psychological Society (2004, CIII). Participants provided written informed consent that their data would be anonymously (i.e., without access to their names) saved, analysed, and published.

### Apparatus, stimuli and procedure

**Fig. 1:**
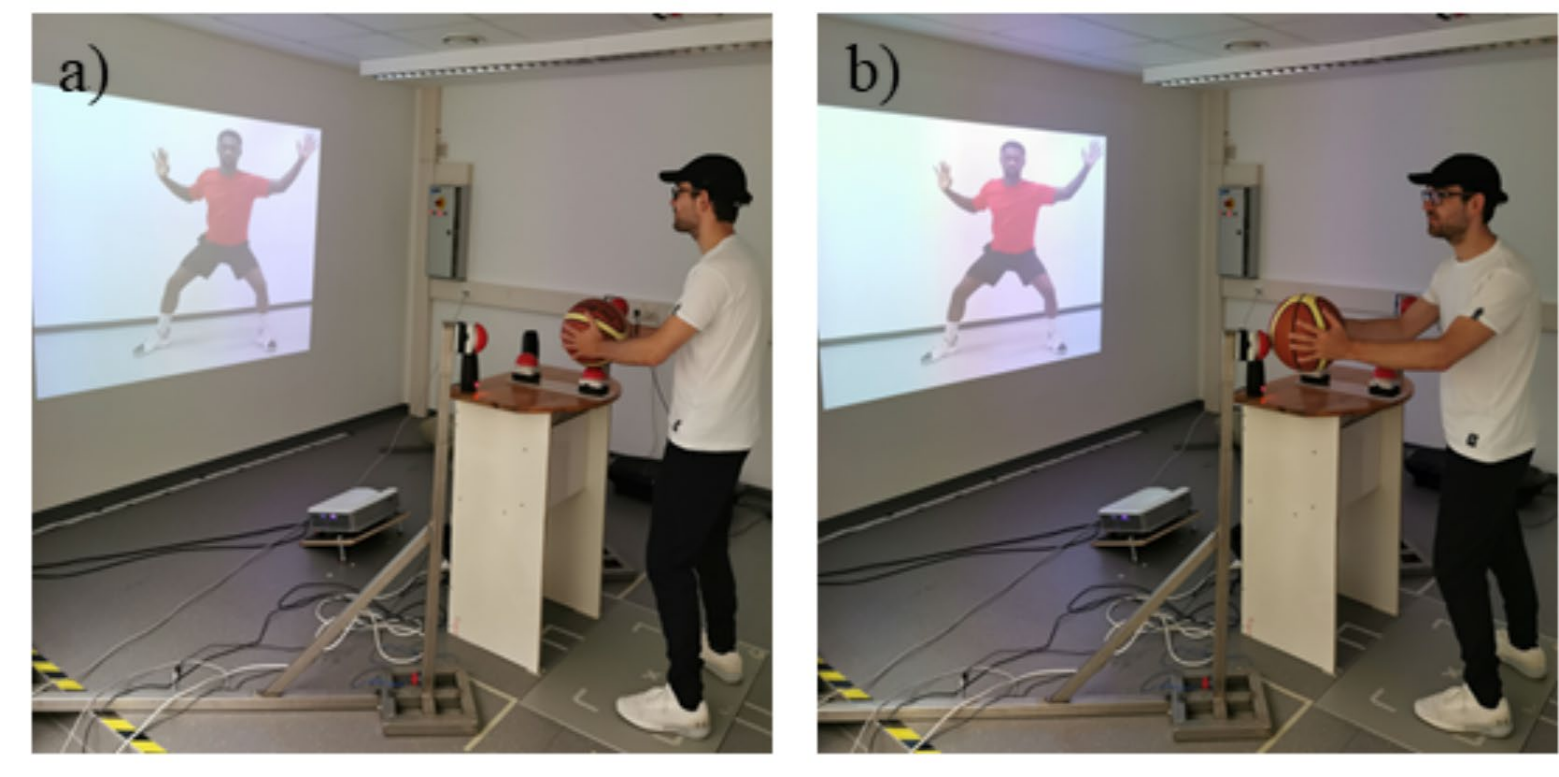
Setup of the Experiment. Setup of the experiment, exemplarily shown for a visual stimulus of a basketball player who covers the right side (from the observer’s perspective). Therefore, the participant has to imitate a passing movement to the left side. Here, the red jersey signals that a passing movement without head fake has to be performed. Picture **a** shows the participant with the basketball on the starting position waiting for the auditory GO signal. Picture **b** shows the end of the pass without head fake to the left side with the participant pressing the left button with the basketball

Participants stood 250 cm away from a screen wall, positioned at a purpose-built apparatus engineered for executing and measuring basketball passes, both with and without a head fake. The apparatus consisted of two steel holders flanking a desk, each equipped with buttons (height: 1.20 m; button separation: 1.25 m). To signify a pass to the left or right, participants utilized a basketball to depress these buttons. Additionally, a button on the desk in front of participants (height: 1 m; cf. Figure 1) was incorporated.

The static visual stimuli featured two distinct basketball players, one clad in a red shirt and the other in blue. The depictions captured the players executing a defensive manoeuvre towards one side, obstructing a potential pass with their body orientation and a raised hand on that side. Conversely, the opposite side remained unguarded, implying an opportunity for a pass (cf. Figure 1). These visual stimuli were projected onto a screen wall (height: 140 cm, width: 200 cm) before the participants, employing an Optoma X320 projector. On the first day of practice, participants were shown four brief videos of a professional basketball player executing a pass with or without a head fake to the left or right side. This was done to familiarize them with the movements they would be required to perform. Subsequently, participants were handed a basketball and instructed to place it onto a button on the desk in front of them, referred to henceforth as the starting position. Additionally, participants were equipped with a black cap adorned with a white stripe along the middle of the visor (posterior to anterior). This cap, coupled with a camera positioned above the participants' starting point, facilitated the analysis of head movements for each trial. Participants were then instructed to perform a passing action to the side that was not defended by the basketball player presented in the visual stimuli. The pass, contingent on the color of the basketball player's shirt, was to be executed with or without a head fake. Participants received instructions to initiate both head and basketball movements simultaneously for both pass types. The allocation of passes with or without head fakes to the corresponding shirt colors was counterbalanced across participants. Execution of the pass was to occur only upon the presentation of an auditory GO-signal (300Hz, jigsaw soundwave), with participants initiating the passing action only after planning their reaction. This GO-signal was either presented simultaneously with the visual cue (ISI 0 ms) or 400 ms, 800 ms or 1200 ms after the stimulus onset.

**Fig. 2:**
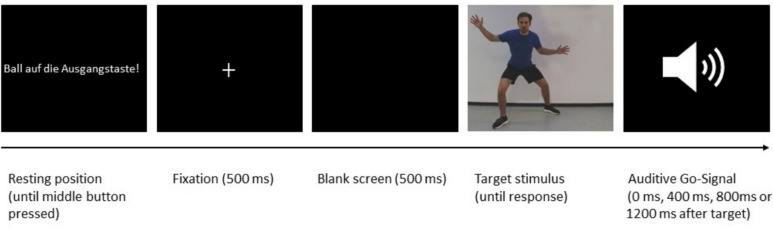
Trial Sequence. Each trial started with the instruction to place the basketball on the start button (“Ball on start button”). A white fixation cross appeared, which was followed by a blank screen. Afterwards, the target stimulus of the basketball player was displayed, until the participants gave their response after the auditory Go-signal. The auditive cue was played either directly with the onset of the target (0 ms) or with a short delay (400 ms, 800 ms, or 1200 ms). The participants responded by either performing a pass with or without head fake to the left or right and pressing the corresponding response button with the basketball. In this example, participants would have to perform a pass (with or without head fake, depending on the assignment of the *type of pass* to the shirt color) to the left “open” side after the auditive cue was played

Each trial initiated with an instruction displayed on the screen, prompting the participant to place the basketball on the start button. This action signalled the commencement of the respective trial. Initially, a white fixation cross appeared for 500 ms at the centre of the screen, succeeded by a 500 ms blank screen before the display of the basketball player target stimulus. The target stimulus persisted on the screen until participants utilized the basketball to press one of the response buttons (on the left or right side of the basketball apparatus). In case participants responded before the auditory GO-signal, the German words “Zu schnell” (too fast) were presented for 1000 ms. Following the participants' passing movement to the left/right, the instruction to place the basketball on the start button reappeared, marking the commencement of the next trial. The trial sequence is illustrated in Fig. 2.

On the first day of practice, participants began the experiment with a practice block of 32 trials in a fixed order and received feedback on their pass direction and head movement after each trial from the instructor. If a participant struggled with performing the correct head turning and passing movement even at the end of the practice block (4 or more wrong answers in the last 8 trials), the participant had to complete the practice block again. After the practice block was completed, the participants performed 4 training blocks of 80 trials each. The trials varied with regard to *type of pass* (pass with head fake vs. pass without head fake) and *ISI* (0 ms, 400 ms, 800 ms, 1200 ms), and were presented in randomized order. Thus, each condition was repeated ten times within a block. The direction of the pass (i.e., left vs. right) was evenly distributed and not manipulated as an experimental factor. On the second through fifth days of training, participants of the practice group exclusively undertook a brief practice block of 8 trials in a fixed order before engaging in the four subsequent training blocks of 80 trials each. Participants of the control group did not practice on days 2, 3 and, 4 but performed the same four blocks of 80 trials each at day 5.

### Data analysis part I

Part I is dedicated to the analysis of mixture effects (Miller, 2006) in ITs to evaluate if practicing the task affects performance on an individual level. IT is the time interval between presentation of the GO-signal and the point in time when participants lifted the ball from the starting position. The data for this analysis is taken from the study of Böer et al. (2024). An overview of the ITs, separately for the practice and the control group, in dependence of *day*^1^ (day 1, day 5), *ISI* (0 ms, 400 ms, 800 ms, 1200 ms), and *type of pass* (pass with head fake, pass without head fake) is shown in Fig. 5 (section Results part II; Comparison of the mean ITs between the practice and the control group).

While Miller’s MixTest does not require a specific distribution for the reaction times, it is designed to work with the typical right-skewed distributions observed in RT/IT data, which are often well-described by an ex-Gaussian distribution (Miller, 2006; Whelan, 2008). Ex-Gaussian distributions are a mixture of a normal distribution and an exponential distribution. Before computing the MixTest, we first evaluated whether a Gauss function or an ex-Gauss function describes the IT data better (see section below). By doing so, we also extracted the values for the probability distribution family (here ex-Gaussian distribution), of which at least estimates for the parameter values mu, sigma, and tau were required to run the MixTest.

#### Analysis of the IT distribution fit

**Fig. 3:**
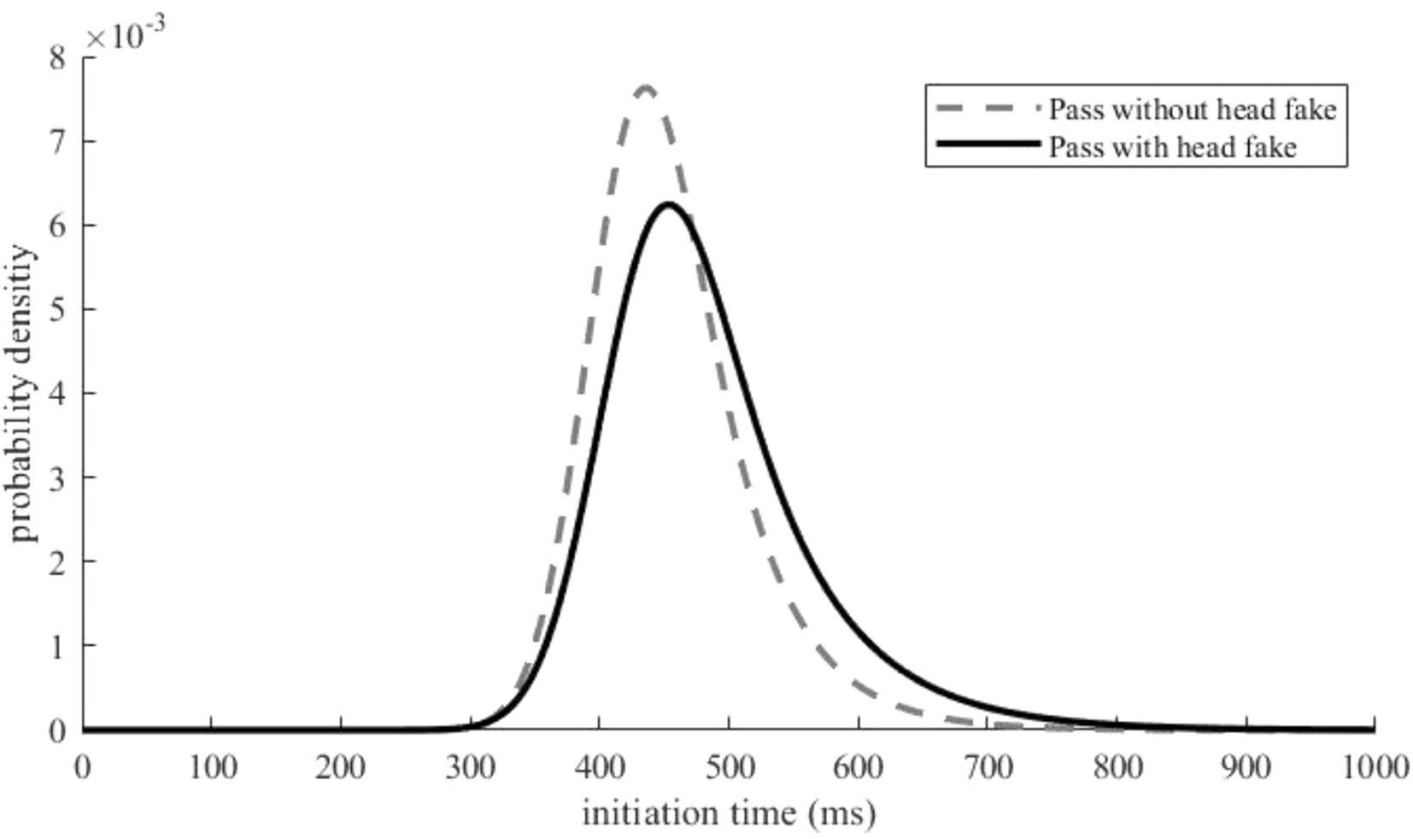
Distribution of Initiation Times at the First Day of Practice. Distributions of ITs the first day of practice for passes without head fakes and passes with head fakes obtained from averaging individual parameter estimates

**Fig. 4:**
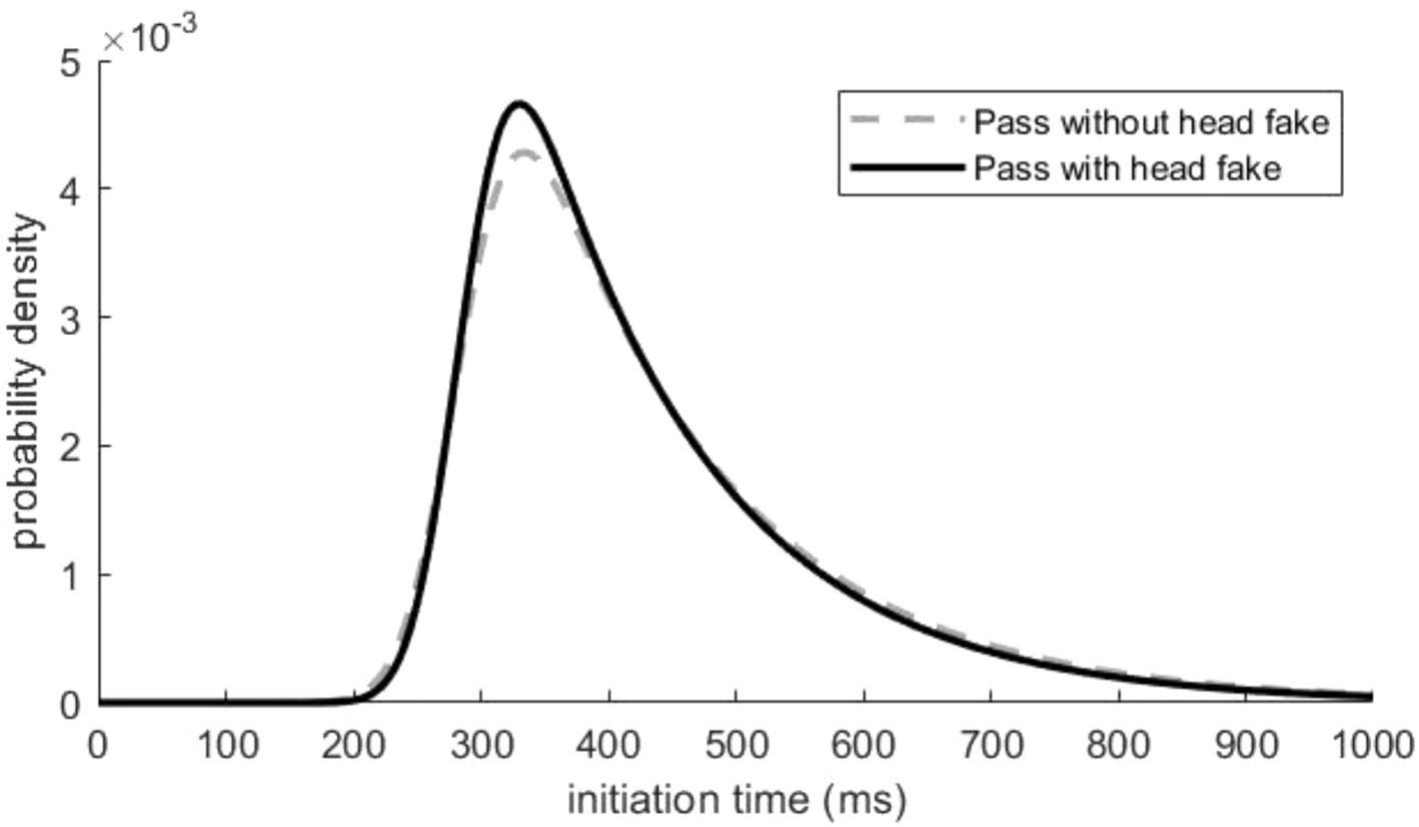
Distribution of Initiation Times Averaged Over all Five Days of Practice. Distributions of ITs averaged over all days of practice for passes without head fakes and passes with head fakes obtained from averaging individual parameter estimates

To evaluate whether a Gauss function or an ex-Gauss function describes the IT data better, we first calculated Akaike’s information criterion (AIC, cf. Lacouture & Cousineau, 2008) for all participants of the practice group. This relatively stable goodness of fit parameter was calculated both for the ex-Gauss model and for the Gauss model. The distribution of passes with and without head fakes of the averaged ITs across participants for the first day of practice is provided in Fig. 3. The averaged distributions over all days of practice and ISIs for passes with and for passes without head fakes can be found in Fig. 4 and illustrate a visible skew in the distribution, which is more pronounced for passes with head fakes (i.e., higher values for tau). Accordingly, the initiation time frequency distribution for passes with and without head fakes of each participant were fitted to an ex-Gaussian distribution using a MATLAB routine (cf. Lacouture & Cousineau, 2008) for each *day * (day 1, day 2, day 3, day 4, and day 5) and each *ISI* (0 ms, 400 ms, 800 ms, 1200 ms). The calculated scores of each participant for the fit for the ex-Gaussian and the Gaussian function were aggregated over the factors *day*, *ISI*, and *type of pass* before the fit was compared using a paired samples t-test. The analysis of the AIC revealed significantly better fitting values for the ex-Gaussian function (19,520.833) than for the Gaussian function (110,137.883) (*t*(23) =  – 5.567, *p* < 0.001, *d* = -1.136).

#### Analysis of mixture effects of the practice group

The IT distribution was examined for mixture effects, as outlined by Miller (2006), utilizing the MixTest program developed by the same author. This program is used to analyse reaction time distributions and determine whether participants show uniform effects of an experimental condition (e.g., slower reaction times in all incongruent trials of the Stroop paradigm compared to congruent trials) or mixed effects (e.g., some incongruent trials with very slow reaction times and some trials without any difference to congruent trials). We used the MixTest to assess whether the production costs of passes with a head fake result in a consistent slowing of all trials involving the production of a head fake (uniform model), or from a combination of some slowed trials with some that are not slowed, suggesting that the fake-production costs originate from only a subset of trials (mixture model). The likelihood ratio test thus compares the two competing models (uniform vs. mixed). As the MixTest program assumes that the experimental condition (here: passes with head fakes) has a higher cognitive load than the control condition (here: passes without head fakes) it cannot calculate mixture effects for cases where the mean IT of the experimental condition is lower than of the control condition. This is referred to as a ‘bad’ effect in the analysis, indicating that there might be no consistent difference between both conditions.

We only calculated the likelihood ratio test for the data from the ISI 0 ms and the ISI 400 ms, as our previous analysis of the mean ITs only revealed fake-production costs at those ISIs but not for the longer ones. The test was run separately for both the ISI 0 ms and the ISI 400 ms as the fake-production costs differed significantly between these ISIs and practice seems to affect these cognitive costs differently.

In the uniform model, ITs from the control condition (pass without head fake) and the experimental condition (pass with head fake) are derived from two distinct IT distributions. The parameters of the model are the parameters of the distribution (for instance, mu, tau, and sigma for an ex-Gaussian distribution). The mixture model also has two distinct IT distributions; however, a proportion of ITs in the experimental condition is unaffected by the experimental manipulation, and thus, comes from the same distribution as the control condition. The experimental effect is only present in the remaining proportion of affected trials and their ITs come from the other, "effect-present", IT distribution. The parameter of the mixture model, which describes the proportion of affected trials, is *P*. A *P*-value of 1 thus indicates that the experimental effect (here fake-production costs) is present in all trials, and thus, reflects a uniform effect. A lower *P*-value, in contrast, indicates that some of the initiation times to perform passes in basketball are not affected. This pattern might reflect a mixed effect, as the fake-production costs only come from a proportion *P* of affected trials (see Miller, 2006 for more details).

It's worth noting that the uniform model is a special case of the mixture model, specifically with 100% affected experimental trials (i.e., *P* = 1.00). A chi-square likelihood ratio test is used to decide whether the mixture model fits significantly better than the uniform model. A significant test (i.e., the significance level of the observed chi-square value) indicates a mixture effect and, thus, that the null hypothesis of a uniform effect needs to be rejected.

## Results part I

### Mixed effect analyses of the practice group

Table 1 summarizes the results of the mixture analysis for the ISI of 0 ms calculating whether there was a uniform or significant mixture effect. For 14 of 24 participants (participants 2, 5, 6, 9, 10, 12, 13, 14, 18, 19, 20, 21, 22, 23), the assumption of a uniform effect could not be rejected (see Table 1). The other 10 participants (1, 3, 4, 7, 8, 11, 15, 16, 17, 24) showed statistically significant mixture effects by the likelihood ratio test aggregated across all sessions. None of the participants consistently exhibited mixture effects across all practice days and the number of participants showing a mixture effect even reduced from day 1 to day 5 (4, 3, 4, 2, 0). Interestingly some participants did not show fake-production costs in some sessions, with participants 7, 14 and 23 having higher ITs for passes without than for passes with head fakes on multiple days of practice.

**Table 1:**
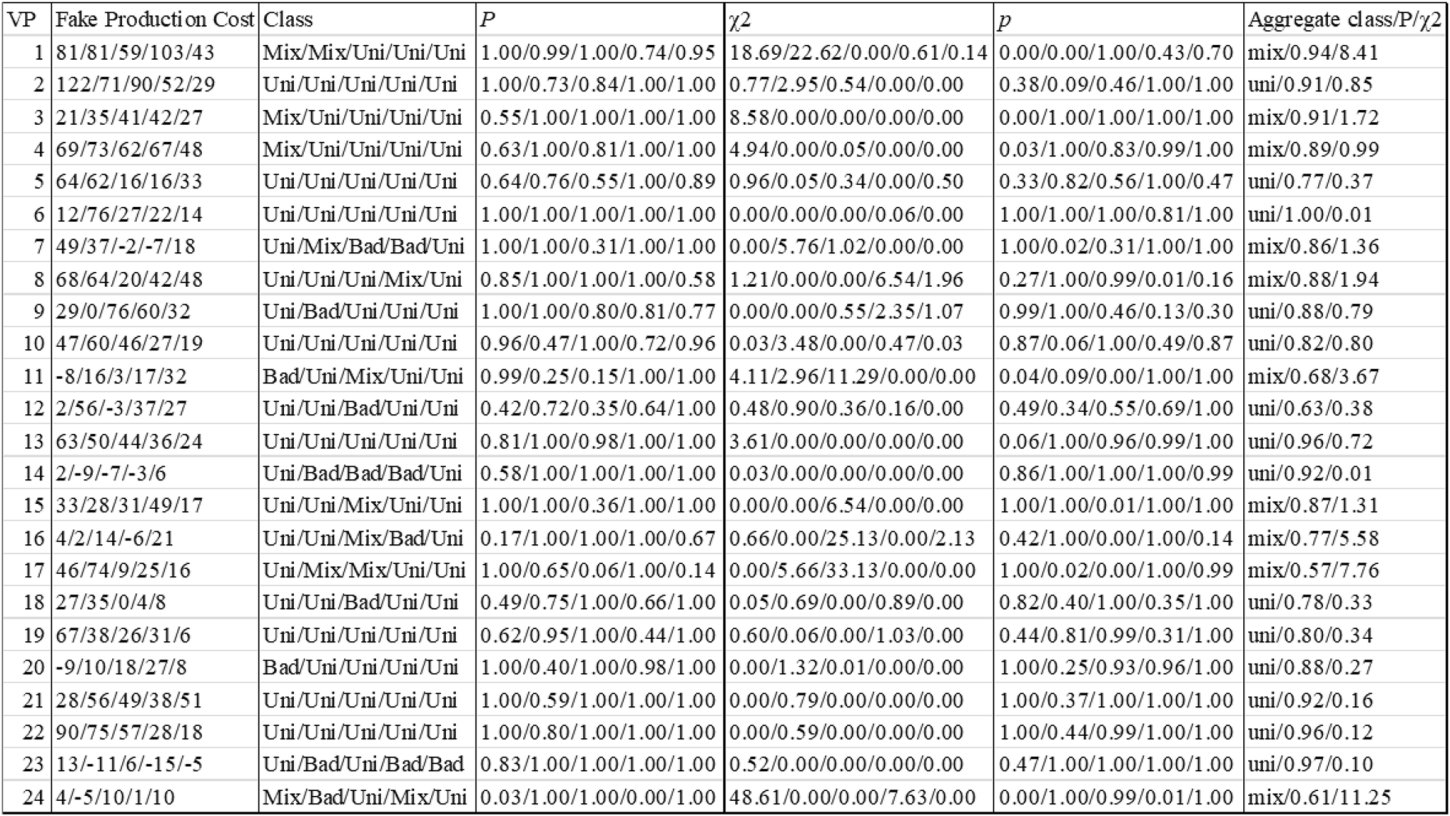
Analysis of Mixture Effects for ISI 0 ms

The values are provided separately for each day of practice (days 1–5). In the column on the most right, the aggregated values across all sessions are provided. Presented are from left to right: Observed fake-production costs (in ms), class of the effect, estimated mixture proportion (*P*), observed *χ2* value of the likelihood ratio test, and corresponding *p* value. Aggregate *P* is the mean *P* across the days of practice. Aggregate *χ2* is the total *χ2* across all days of practice. Each observed *χ2* has one degree of freedom, and the aggregate *χ2*s have five degrees of freedom

The mixture model analysis also provides an estimate of the proportion of the experimental trials in which participants showed fake-production costs, the mixture proportion (*P*, see Table 1). Participants 11, 12, 17, and 24 had an aggregated mixture proportion below 0.75, which means that they showed fake-production costs in only 68%, 63%, 57%, and 61% of the times, respectively, when they performed a pass with a head fake at an ISI of 0 ms. All other participants have an aggregated mixture proportion > 0.75, which means that the majority of participants showed fake-production costs slowing their ITs down in over 75% of trials where they played a pass with compared to a pass without a head fake.

Table 2 summarizes the results of the mixture analysis for the ISI of 400 ms. Here for only 8 of 24 participants (participants 4, 7, 9, 10, 14, 17, 19, 23), the assumption of a uniform effect could not be rejected (see Table 2). One participant did not show fake-production costs at any day of practice at the ISI 400 ms and therefore the mixture analysis could not analyse the data properly (no categorization to mixture or uniform effect possible). The other 15 participants (1, 2, 3, 4, 5, 6, 11, 12, 13, 15, 16, 18, 20, 21, 22, 24) showed statistically significant mixture effects by the likelihood ratio test aggregated across all sessions. None of the participants consistently exhibited mixture effects across all practice days and the number of participants showing a mixture effect did not change greatly from day 1 to day 5 (6, 6, 5, 7, 5). The analysis also revealed an increasing number of bad effects with increasing practice from day 1 to day 5 (3, 7, 11, 12, 12). This increase in bad effects in the mixture model indicates that there might not be a consistent difference between passes with and without head fakes at the ISI 400 ms since an increasing number of participants showed bad effects, meaning they were able to overcome the fake-production costs.

**Table 2:**
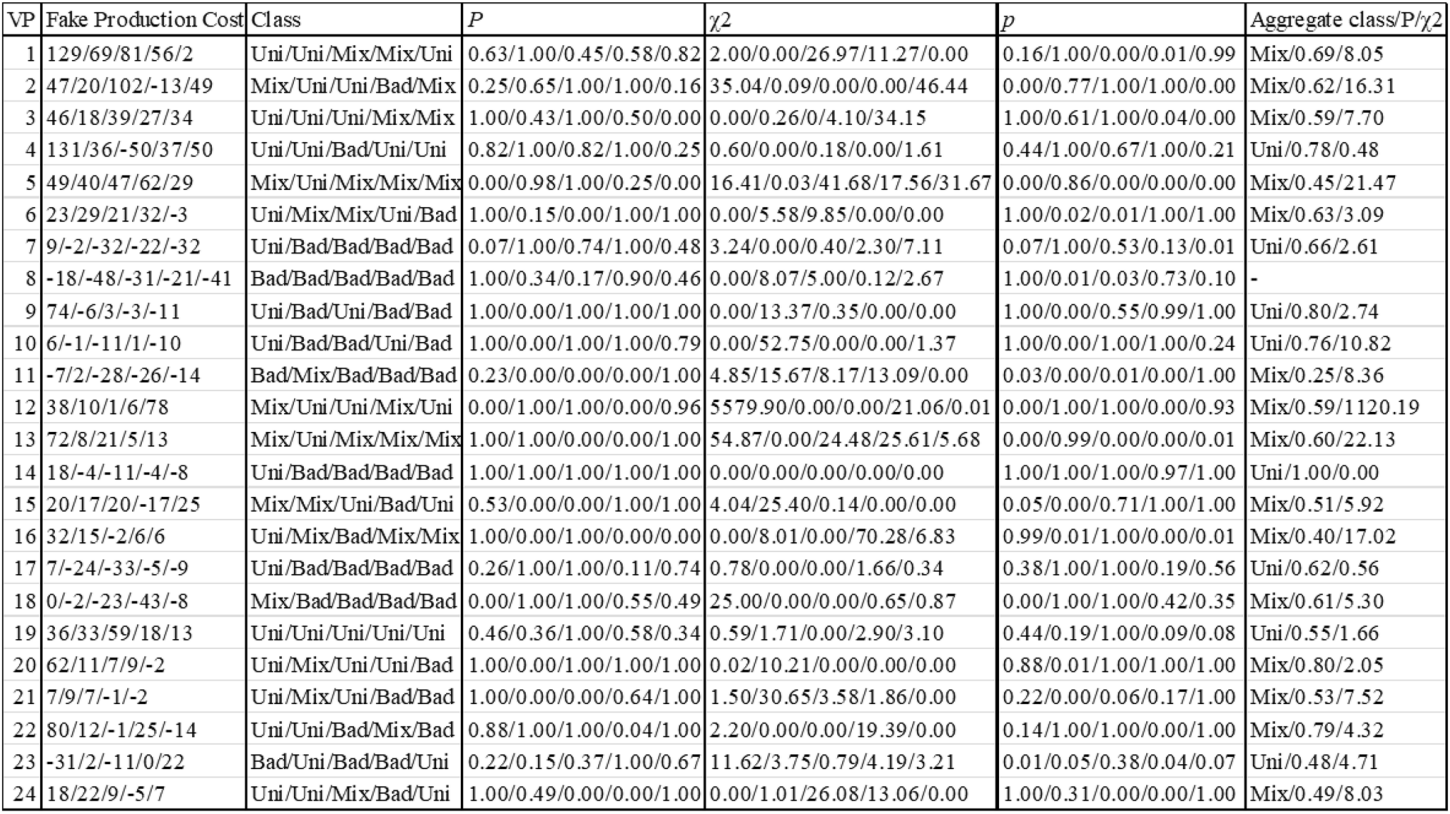
Analysis of Mixture Effects for ISI 400 ms

The values are provided separately for each day of practice (days 1–5). In the column on the most right, the aggregated values across all sessions are provided. Presented are from left to right: Observed fake-production costs (in ms), class of the effect, estimated mixture proportion (*P*), observed *χ2* value of the likelihood ratio test, and corresponding *p* value. Aggregate *P* is the mean *P* across the days of practice. Aggregate *χ2* is the total *χ2* across all days of practice. Each observed *χ2* has one degree of freedom, and the aggregate *χ2*s have five degrees of freedom

The mixture model analysis also provides an estimate of the proportion of the experimental trials in which participants showed fake-production costs, the mixture proportion (*P*, see Table 2). Here, 17 participants had an aggregated mixture proportion below 0.75, which means most participants showed fake-production costs in under 3 out of 4 cases. The other 6 participants have an aggregated mixture proportion > 0.75, which means they did show fake-production costs slowing their initiation times down in over 75% of trials where they played a pass with compared to a pass without a head fake.

Overall, these findings suggest that mental preparation time significantly impacts whether fake-production costs occur consistently or sporadically across trials. The short preparation time at ISI 400 ms seemed to be sufficient to reduce the percentage of trials in which participants elicited fake-production costs, with some even overcoming them. In contrast, without mental preparation time (ISI 0 ms) participants showed consistent fake-production costs even on day 5.

### Data analysis part II

Part II focusses on the comparison of mean ITs and ex-Gaussian distribution parameters of the practice group with the newly collected control group to analyse whether the improvement of participants’ ITs in the practice group (cf. Böer et al., 2024) were based on the effects of practicing the passes or merely reflected test–retest effects.

#### Analysis of the mean ITs of the practice and the control group

IT was the time interval between presentation of the GO-signal and the point in time when the participant lifted the ball from the starting position. The alpha-level chosen for significance was 0.05. A violation of the sphericity-assumption resulted in a correction of the *p*-values according to Greenhouse–Geisser. Partial eta-squared (ɳ_p_^2^) values of 0.01, 0.06, and 0.14 were interpreted to indicate small, medium, or large effects, respectively (Richardson, 2011).Trials were excluded if the IT deviated by more than three standard deviations from the cell mean, calculated separately for each participant, *ISI*, *type of pass*, and *day * (1.6% of trials in the practice group, 1.4% of trials in the control group) and if participants moved incorrectly (too early or to the wrong side; 3.6% of trials in the practice group, 4.3% of trials in the control group). Participants’ ITs were analysed with a repeated measure ANOVA with the factors *type of pass* (pass without head fake, pass with head fake), *ISI* (0 ms, 400 ms, 800 ms, 1200 ms), *day* (day 1, day 5), and the between-subjects factor *group* (practice group, control group). Before performing the statistical analysis, the normal distribution was checked with the Shapiro–Wilk test for all analysed data and homoscedasticity with Levene’s test. Group differences in practice-related reductions in mean ITs were analysed with post-hoc comparisons, in which *p*-values were all pairwise post-hoc corrected (adjusted to Holm-Bonferroni; Holm, 1979).

#### Analysis of the ex-Gaussian parameters of the practice and the control group

We used a modified version of publicly available Matlab code (Lacouture & Cousineau, 2008) to estimate the ex-Gaussian distribution parameters mu, sigma, tau (*μ, σ, τ*) and fit the ex-Gaussian distribution to the IT data using maximum likelihood estimations.

The parameters mu, sigma and tau of the ex-Gaussian distribution were then analysed in relation to the independent variables *type of pass* (pass with head fake, pass without head fake), *day* (day 1, day 5), *ISI* (0 ms, 400 ms, 800 ms, 1200 ms), and the between-subjects factor *group* (control group, practice group).

## Results part II

### Comparison of the mean ITs between the practice and the control group

**Fig. 5:**
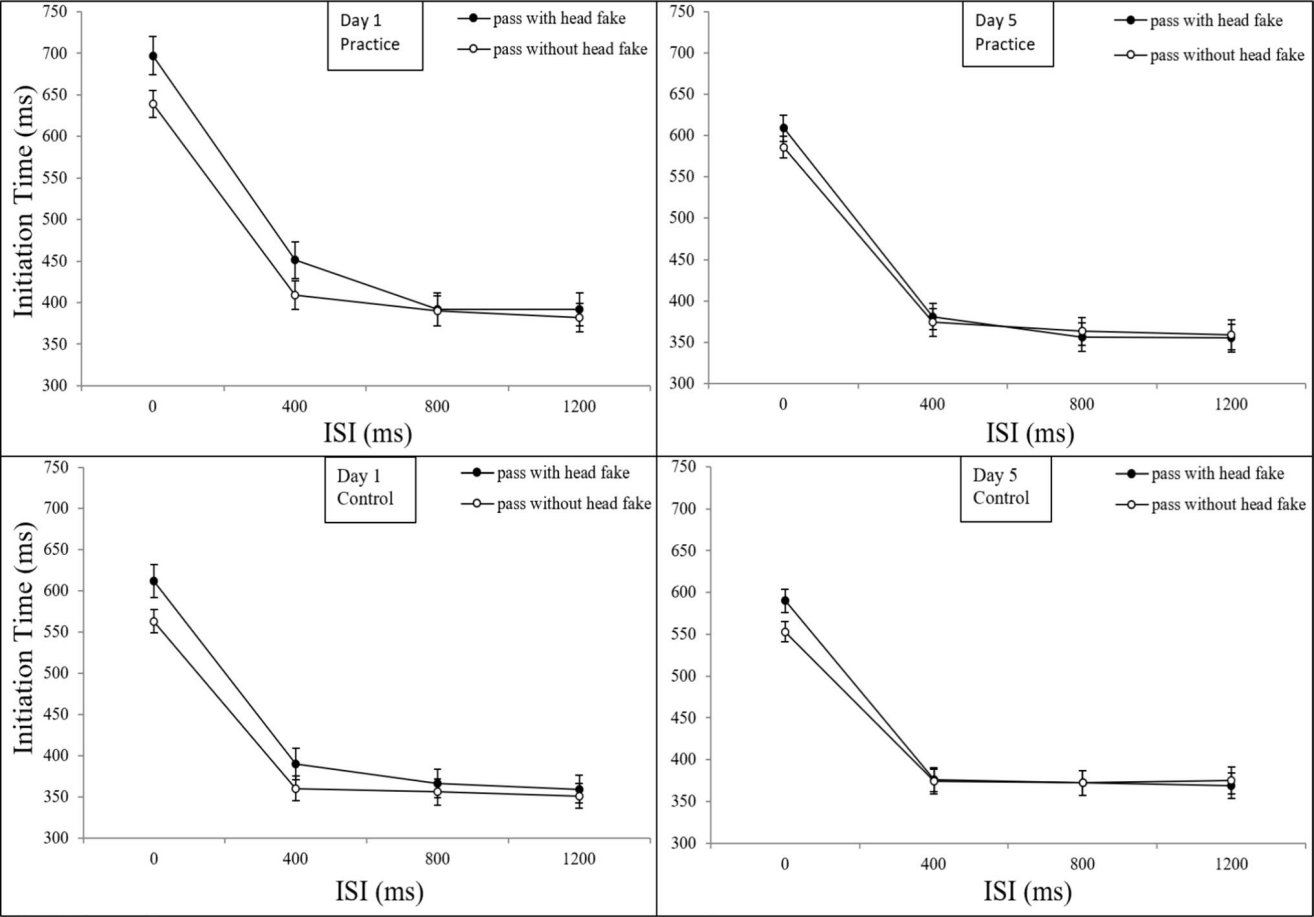
ITs from Day 1 and Day 5 as a Function of Type of Pass for both Groups. Mean initiation times (ITs) for day 1 and day 5, separated for the practice and the control group, as a function of *type of pass* (error bars show standard errors)

A mixed ANOVA with mean IT as dependent variable and the between-subjects factor *group* (control group, practice group) and the within-subjects factors *type of pass* (pass without head fake, pass with head fake), *day* (day 1, day 5), and *ISI* (0 ms, 400 ms, 800 ms, 1200 ms) revealed a main effect for the factor *t*y*pe of pass*: initiation times were significantly slower for passes with head fakes (*M* = 442 ms) than for passes without head fakes (*M* = 425 ms), *F*(1,54) = 20.16; *p* < 0.001; *ɳ*_*p*_^*2*^ = 0.272*; ε* = 0.993. Also, the ANOVA indicated a main effect for the factor *ISI*, pointing to a reduction of initiation times with increasing *ISI* length, *F*(1,54) = 1102.07; *p* < 0.001; *ɳ*_*p*_^*2*^ = 0.953*; ε* = 1. The factor *day* also reached significance*,* with decreasing mean ITs from day 1 (*M* = 444 ms) to day 5 (*M* = 423 ms), *F*(1,54) = 4.81; *p* = 0.033; *ɳ*_*p*_^*2*^ = 0.082*; ε* = 0.577. We found significant interactions between the factors *day* and *ISI*, *F*(3,162) = 20.94; *p* < 0.001; *ɳ*_*p*_^*2*^ = 0.279*; ε* = 1, between *type of pass* and *ISI*, *F*(3,162) = 56.06; *p* < 0.001; *ɳ*_*p*_^*2*^ = 0.509*; ε* = 1, as well as an interaction between *type of pass* and *day*, *F*(1,54) = 19.85; *p* < 0.001; *ɳ*_*p*_^*2*^ = 0.269*; ε* = 0.992. The ANOVA also revealed a three-way interaction between *type of pass*, *ISI*, and *day*, *F*(3,162) = 5.04; *p* = 0.002; *ɳ*_*p*_^*2*^ = 0.085*; ε* = 0.913, see Fig. 5.

To analyse the three-way interaction of the factor *day* with the factor *type of pass* and the factor *ISI* we performed paired *t*-tests of the changes in ITs from day 1 to day 5. The analysis indicated a significant reduction of ITs for passes with head fakes at the ISI 0 ms from day 1 (*M* = 648 ms) to day 5 (*M* = 600 ms), *t*(55) = 3.976, *p* = 0.002, *d* = 0.531, as well as for passes without head fakes from day 1 (*M* = 596 ms) to day 5 (*M* = 567 ms), *t*(55) = 3.548, *p* = 0.005, *d* = 0.474. Also, we found a significant reduction of ITs for passes with head fakes at the ISI 400 ms from day 1 (*M* = 416 ms) to day 5 (*M* = 378 ms), *t*(55) = 2.860, *p* = 0.036, *d* = 0.382. All other comparisons did not reach significance (*p* > 0.05).

We further evaluated how fake-production costs (passes with head fakes minus passes without head fakes) changed at ISI 0 ms and at ISI 400 ms from day 1 to day 5. Paired *t*-tests showed that fake-production costs significantly decreased at ISI 0 ms from day 1 (*M* = 52 ms) to day 5 (*M* = 33 ms), *t*(55) = 2.803, *p* = 0.007, *d* = 0.375, and at ISI 400 ms from day 1 (*M* = 35 ms) to day 5 (*M* = 4 ms), *t*(55) = 4.824, *p* < 0.001, *d* = 0.645.

Regarding the main factor of interest, namely the between-subjects factor *group*, there was no main effect (*p* > 0.05). However, there were significant interactions between the factors *group* and *ISI*, *F*(3,162) = 8.84; *p* < 0.001; *ɳ*_*p*_^*2*^ = 0.141*; ε* = 0.995, modulated by the generally higher ITs of the practice compared to the control group at ISI 0 ms (53 ms) and at ISI 400 ms (29 ms) which wasn’t of further interest for our study (see Fig. 5) and between the factors *group* and *day*, *F*(1,54) = 6.53; *p* = 0.013; *ɳ*_*p*_^*2*^ = 0.108*; ε* = 0.709, indicating group differences in the improvement of ITs from day 1 to day 5.

**Fig. 6:**
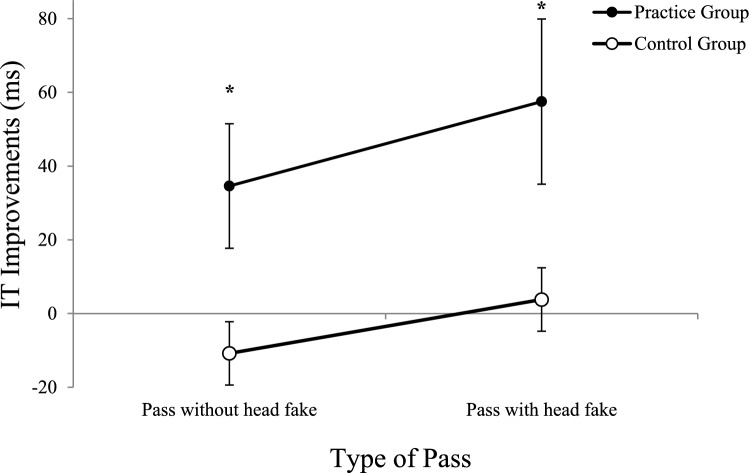
Improvements in IT from Day 1 to Day 5 as a Function of Group and Type of Pass. Mean reduction of initiation times (IT) from day 1 to day 5 as a function of *type of pass* and *group* (error bars show standard errors of the mean differences of passes with and without head fakes per day). Negative values indicate an increase in initiation times. Asterisks indicate significant differences between groups

To evaluate the differences in improvements of ITs between day 1 and day 5 between the practice and the control group a post-hoc *t*-test was conducted. First, the overall changes in the groups ITs (aggregated over the factors *ISI* and *type of pass*) were calculated by subtracting the IT of day 5 from the IT of day 1. Then the changes in ITs between both groups (see Fig. 6) were compared by an independent samples *t*-test, which revealed significantly higher reductions of ITs for the practice (*M* = 46 ms) compared to the control group (*M* =  – 3 ms), *t*(54) =  – 2.557, *p* = 0.013, *d* =  – 0.691. More precisely, further *t*-tests (one-sample *t*-test for each group) were conducted to determine whether mean IT improvements from day 1 to day 5 differed significantly from zero. The one-sample *t*-test for the IT improvement of the practice group (*M* = 46 ms, *SD* = 95 ms) showed a significant difference from zero, *t*(23) = 2.375, *p* = 0.026, *d* = 0.485, while the control group showed no significant differences from zero (*p* > 0.05).

Taken together, the post-hoc analyses of the interaction between *day*, *type of pass,* and *ISI* indicated reduced fake-production costs at ISI 0 ms and at ISI 400 ms. The interaction between the factors *group* and *day* showed that only the practice group improved their performance significantly from day 1 compared to day 5. In contrast, the control group showed no change between their performance on day 1 and day 5. We therefore suggest that, although there was no four-way interaction with group, reduced fake-production costs from day 1 to day 5 are driven by practice and not solely by test–retest effects. We further analyse this not entirely clear data pattern in the later sections.

### Comparison of the ex-Gaussian parameters between the practice and the control group

The results of the analysis of the mean ITs indicate that practice leads to a general reduction of ITs, but it is unclear whether practice affects passes with head fakes in a greater extent than passes without head fakes. To gain a more comprehensive understanding of potential differences in cognitive processing through practice between both groups, we conducted an ex-Gaussian analysis of IT distributions in the next section. This approach allows for a more nuanced examination of the IT data than focusing solely on measures of central tendency. Therefore, mixed ANOVAs with the factors *day* (day 1, day 5), *ISI* (0 ms, 400 ms, 800 ms, and 1200 ms), *type of pass* (pass without head fake, pass with head fake), and the between-subjects factor *group* (control group, practice group) for the distribution parameters mu, sigma, and tau were performed.

### Distribution mean (Mu)

An ANOVA with the distribution mean (mu) as dependent variable, the between-subjects factor *group* (control group, practice group), and the within-subjects factors *type of pass* (pass without head fake, pass with head fake), *day* (day 1, day 5), and *ISI* (0 ms, 400 ms, 800 ms, 1200 ms) was conducted. A significant main effect was observed for the factor *ISI*, *F*(3, 162) = 841.14; *p* < 0.001; *ɳ*_*p*_^*2*^ = 0.940; *ε* = 1, but no main effects were found for *day*, *type of pass, * and for the between-subjects factor *group* (all *p* > 0.05).

However, the interaction of *ISI* with *day*, *F(*3,162) = 15.25; *p* < 0.001; *ɳ*_*p*_^*2*^ = 0.220*; ε* = 0.995, as well as the interaction of *ISI* with *type of pass*,* F*(3,162) = 26.54; *p* < 0.001; *ɳ*_*p*_^*2*^ = 0.330*; ε* = 1, reached significance. Additionally, a significant interaction was observed between the factors *type of pass* with *day*, *F(*1,54) = 7.49; *p* < 0.01; *ɳ*_*p*_^*2*^ = 0.122*; ε* = 0.767, as well as a significant three-way interaction between *type of pass*, *day,* and *ISI*, *F(*3,162) = 6.52; *p* < 0.01; *ɳ*_*p*_^*2*^ = 0.108*; ε* = 0.921.

**Fig. 7:**
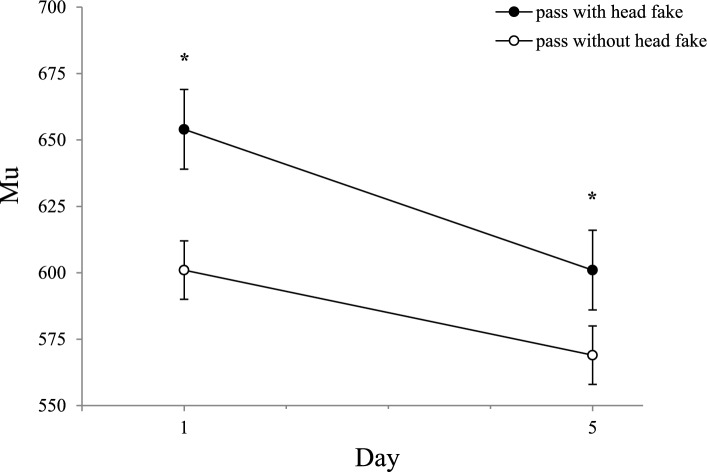
Mu as a Function of Day and Type of Pass for ISI 0 ms. Mean mu values as a function of *day* and *type of pass* for the ISI 0 ms (error bars show standard errors of the mean). Asterisks indicate significant differences between passes with and without head fake

To analyse the three-way interaction of the factor *day* with the factor *type of pass* and the factor *ISI* we performed paired *t*-tests of the changes in mu from day 1 to day 5. The analysis indicated a significant reduction of mu for passes with head fakes at the ISI 0 ms from day 1 (*M* = 591) to day 5 (*M* = 544), *t*(55) = 3.944, *p* < 0.001, *d* = 0.527, but not for the other pairs (*p* > 0.05). Figure 7 illustrates the changes in mu from day 1 to day 5 for passes with and without head fakes at ISI 0 ms.

We further evaluated if this reduction for head fakes significantly decreased fake-production costs at ISI 0 ms. A paired t-tests showed that fake-production costs significantly decreased at ISI 0 ms from day 1 (*M* = 51 ms) to day 5 (*M* = 16 ms), *t*(55) = 3.727, *p* < 0.001, *d* = 0.498.

The only significant interaction with the between-subjects factor *group* was found with the factor *ISI*, *F(*3,162) = 4.94; *p* < 0.05; *ɳ*_*p*_^*2*^ = 0.084*; ε* = 0.742. Independent sampled *t*-tests of the interaction of the between-subjects factor *group* with the factor *ISI* did not reach significance after the Holm-Bonferroni correction (all *p* > 0.05).

Taken together, the results suggest that the distribution mean (mu) does not reflect the different reductions between the groups found in the mean ITs, as the absence of group differences for mu suggests that the improvements are not enhanced by practice and are likely attributable to test–retest effects. Mu (distribution mean) was significantly higher for passes with head fakes than for those without head fakes at ISI 0 ms. This result highlights the motor programming demands of response-response incompatible movements, when no time is available to mentally prepare the movement.

### Distribution spread (Sigma)

An ANOVA with the distribution spread (sigma) as dependent variable, the between-subjects factor *group* (control group, practice group), and the within-subjects factor *type of pass* (pass without head fake, pass with head fake), *day* (day 1, day 5), and *ISI* (0 ms, 400 ms, 800 ms, 1200 ms) revealed a main effect for the factor *ISI*, *F*(3, 162) = 39.49; *p* < 0.001; *ɳ*_*p*_^*2*^ = 0.422; *ε* = 1.0. The ANOVA also revealed a significant main effect of the factor *day* shown by the reduction of sigma from day 1 (*M* = 38) to day 5 (*M* = 29),* F*(1,54) = 18.19; *p* < 0.001; *ɳ*_*p*_^*2*^ = 0.252*; ε* = 0.987. This reduction indicates less variability in participants’ ITs from day 1 to day 5. The other main effects did not reach significance (*p* > 0.05).

Additionally, we found significant interactions of the factors *ISI* and *day*, *F*(3, 162) = 4.03; *p* = 0.008; *ɳ*_*p*_^*2*^ = 0.069; *ε* = 0.834, of the factors *ISI* and *type of pass*, *F*(3, 162) = 7.60; *p* < 0.001; *ɳ*_*p*_^*2*^ = 0.123; *ε* = 0.986, and of the factors *type of pass* and *day*, *F*(1, 54) = 7.12; *p* = 0.010; *ɳ*_*p*_^*2*^ = 0.117; *ε* = 0.746.

Paired *t*-tests of the interaction of the factor *day* with the factor *ISI* revealed a significant reduction of sigma at the ISI 0 ms from day 1(*M* = 57) to day 5 (*M* = 41), *t*(55) = 4.915, *p* < 0.001, *d* = 0.657, and at the ISI 400 ms from day 1 (*M* = 32) to day 5 (*M* = 21), *t*(55) = 4.206, *p* < 0.001, *d* = 0.562. These results indicate that participants showed less variance in ITs at shorter ISIs as they became more familiar with the task over the course of practice. The other comparisons did not reach significance (*p* > 0.05).

**Fig. 8:**
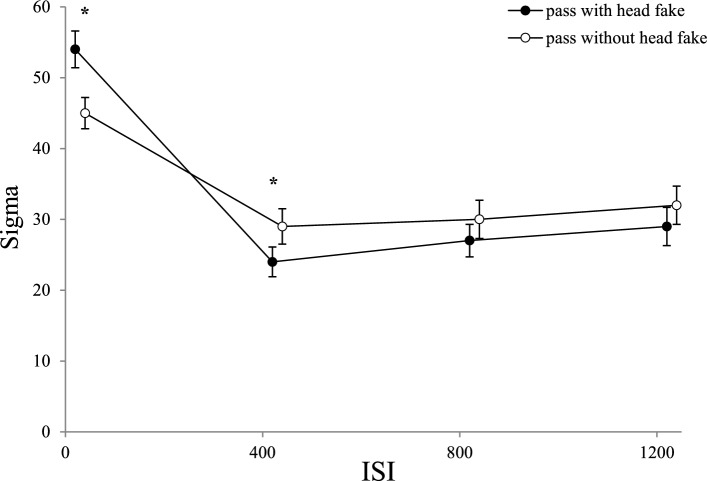
Sigma as a Function of ISI and Type of Pass. Mean Sigma values as a function of *ISI* and *type of pass* (error bars show standard errors of the mean). Asterisks indicate significant differences between passes with and without head fake

Paired *t*-tests of the interaction of the factor *type of pass* with the factor *ISI* revealed a significantly higher distribution spread (sigma) for passes with (*M* = 54) compared to passes without head fakes (*M* = 45) at the ISI 0 ms, *t*(55) = 3.623, *p* < 0.001, *d* = 0.484. This result highlights that fake-production costs increase response variability when participants cannot mentally prepare their movements (see Fig. 8). The other comparisons did not reach significance (*p* > 0.05).

**Fig. 9:**
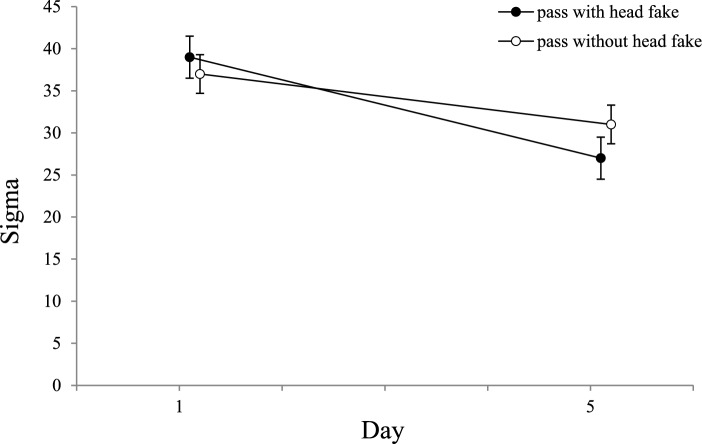
Sigma as a Function of Day and Type of Pass. Mean Sigma values as a function of *day* and *type of pass* (error bars show standard error of mean differences). Horizontal jitter was added to make the plotted data more distinguishable

Furthermore, a single comparison (paired *t*-test) was used to investigate the interaction of the factors *type of pass* and *day*. The difference between day 1 and day 5 were calculated by subtracting the values of day 5 from day 1 for both types of passes (higher values indicating a greater reduction). The post-hoc test revealed a greater reduction of sigma from day 1 to day 5 for passes with head fakes (*M* = 12) than for passes without head fakes (*M* = 5), *t*(55) = 2.670, *p* = 0.010, *d* = 0.357, see Fig. 9.

Similar to the results of the ANOVA for mu, the only significant interaction of the between-subjects factor *group* was with the factor *ISI*, *F*(3, 162) = 3.67; *p* = 0.024; *ɳ*_*p*_^*2*^ = 0.064 *ε* = 0.699. All other interactions did not reach significance (*p* > 0.05). To test the interaction of the between-subjects factor *group* and the factor *ISI*, single comparisons (independent samples *t*-tests adjusted to Holm-Bonferroni) were used. None of the comparisons were significant after correction (*p* > 0.05).

Together, these results suggest that the distribution spread (sigma) reflects a familiarization of participants with the task instead of practice effects, as both groups showed significant reductions of sigma from day 1 to day 5. These findings indicate that one day of practice is sufficient to stabilize performance, allowing participants to respond more consistently. The results also confirm that performing a pass with or without a head fake without any mental preparation time beforehand (ISI 0 ms) is more complex, leading to greater variability in performance compared to longer ISIs.

### Distribution skew (Tau)

An ANOVA with the distribution skew (tau) as dependent variable, the between-subjects factor *group *(control group, practice group), and the within-subjects factors *type of pass* (pass without head fake, pass with head fake), *day* (day 1, day 5), and *ISI* (0 ms, 400 ms, 800 ms, 1200 ms) revealed a significant main effect for the factor *ISI* (*F*(3, 162) = 36.23; *p* < 0.001; *ɳ*_*p*_^*2*^ = 0.402; *ε* = 1). The ANOVA also revealed a main effect for the factor *day,* caused by a reduction of tau from day 1 (*M* = 53) to day 5 (*M* = 40), *F*(1,54) = 11.67; *p* = 0.001; *ɳ*_*p*_^*2*^ = 0.178*; ε* = 0.919. The factor *type of pass* also reached significance, with lower values of tau for passes without (*M* = 39) than for passes with head fakes (*M* = 54)) *F*(1,54) = 14.97; *p* < 0.001; *ɳ*_*p*_^*2*^ = 0.217*; ε* = 0.967.

**Fig. 10:**
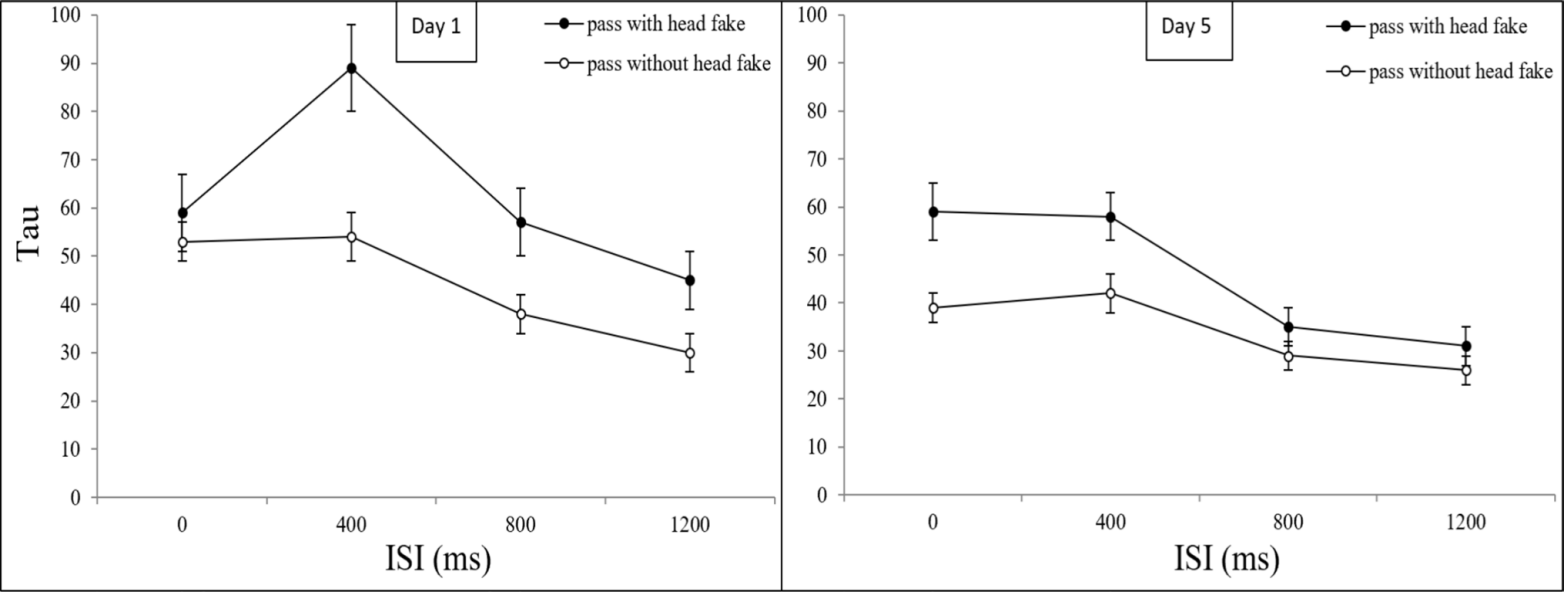
Tau as a Function of Day, Type of Pass and ISI. Mean tau values for day 1 and day 5 as a function of the factors *ISI* and *type of pass* (error bars show standard errors)

The ANOVA also revealed an interaction of *ISI* with *type of pass*,* F*(3,162) = 3.16; p = 0.026; *ɳ*_*p*_^*2*^ = 0.097*; ε* = 0.645, as well as a significant three-way interaction of *ISI* with *day* and *type of pass*, *F*(3,162) = 4.35; p = 0.006; *ɳ*_*p*_^*2*^ = 0.075*; ε* = 0.864 (see Fig. 10).

Paired *t*-tests investigating the three-way interaction of the factors *ISI*, *type of pass*, and *day* revealed only a significant reduction of tau for passes with head fakes at the ISI 400 ms from day 1 (*M* = 87) to day 5 (*M* = 58), *t*(55) = 3.639, *p* < 0.001, *d* = 0.486. No other comparisons were significant (*p* > 0.05).

**Fig. 11:**
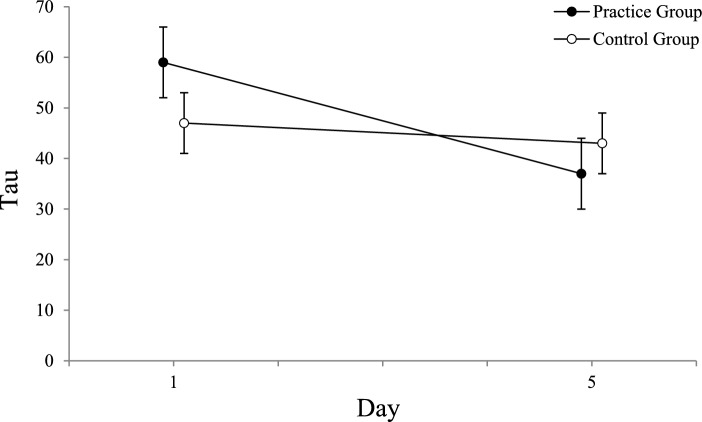
Tau as a Function of Day and Group. Mean values of Tau as a function of the factors *day* and *group* (error bars show standard errors). Horizontal jitter was added to make the plotted data more distinguishable

The only interaction with the between-subjects factor *group*, which reached significance, was with the factor *day*, *F*(1,54) = 5.31; p = 0.025; *ɳ*_*p*_^*2*^ = 0.090*; ε* = 0.619. All other interactions did not reach significance (*p* > 0.05). To analyse the three-way interaction, we calculated the difference of tau from day 1 to day 5 (aggregated over the factors *ISI* and *type of pass*) for both groups. An independent samples t-tests was used to compare these reductions of tau between both groups, which revealed a significantly greater reduction from day 1 to day 5 for the practice group (*M* =  – 21) compared to the control group (*M* =  – 4), *t*(55) =  – 2.304, *p* = 0.025, *d* =  – 0.486, see Fig. 11. Further *t*-tests (one-sample *t*-test for each group) were conducted to determine whether mean improvements of tau from day 1 to day 5 differed significantly from zero. The one-sample *t*-test for the improvement of tau of the practice group (*M* =  – 21) showed a significant difference from zero, *t*(23) =  – 3.210, *p* = 0.004, *d* = -0.655, while the control group (*M* =  – 4) showed no significant differences from zero (*p* > 0.05).

Together the results for the analysis of the distribution skew (tau) revealed higher values of tau for passes with compared to passes without head fakes, indicating increased cognitive demands when performing movements with response-response incompatibility. Tau was the only distribution parameter clearly modulated by practice with the task, with significant reductions in the practice group while the control group did not improve from day 1 to day 5.

## Discussion

The present study was designed to evaluate existing data of an earlier investigation (Böer et al., 2024) with more sophisticated analyses and to overcome some of its limitations. In the study of Böer et al. (2024), it was investigated how physical practice of passes with and without head fakes in a basketball setting modulates the fake-production costs for basketball novices. Participants practiced passes with and without head fakes, performing whole-body movements towards left or right response buzzers on five consecutive days. Similar to other research (Güldenpenning et al., 2023; Kunde et al., 2019), significant fake-production costs were observed when participants had no (ISI 0 ms) or only limited time (ISI 400 ms) to prepare the fake action. Importantly, the study of Böer et al. (2024) reported greater effects of practice for passes with head fakes compared to passes without head fakes, which results in decreased fake-production costs with practice.

In the current manuscript, we first analysed the IT distribution of this practice group for mixture effects to evaluate the effects of practice on an individual level, testing whether participants consistently show fake-production costs in all trials, in which they performed a pass with head fake (uniform effect), or only in some of the trials (mixed effect).

Additionally, a control group was tested ex post, since the original study could not clarify whether the reduction in fake-production costs observed was based on practice or rather reflected test–retest effects. The already collected data of the practice group and the control group were further analysed by calculating ex-Gaussian distribution parameters (mu, sigma, tau) of the initiation times (ITs) to evaluate (1) which parameter(s) of the ex-Gaussian distribution reflect the fake-production costs, and (2) whether practice only leads to a stabilization of performance by decreasing variance (i.e., familiarization; evident in sigma) and/or if practice reduces the number of trials with especially high ITs (i.e., reduction of attentional lapses; evident in tau).

### Implications of the analysis of mixture effects

The analysis of mixture effects (Miller, 2006) for the ISI of 0 ms revealed that most participants (20 of 24) showed a uniform effect for fake-production costs, observed in over 75% of trials where they played a pass with compared to a pass without a head fake. None of the participants showed a consistent mixture effect for all 5 days of practice and the number of participants showing a mixture effect decreased from the first day of practice (4 participants) to the last day of practice (0 participants). This indicates that participants stabilized their performance but also that there are general cognitive costs associated with the production of passes with head fakes without mental preparation time (ISI 0 ms), which cannot be overcome through practice.

In contrast, the analysis for the ISI of 400 ms revealed stronger evidence for a mixed effect as 15 out of 24 participants showed significant mixture effects across the aggregated sessions and most participants (17 of 24) elicited the fake-production costs in less than 75% of trials. It also must be noted that the analysis indicated that the number of participants, who were able to completely overcome the fake-production costs at the ISI of 400 ms, increased from day 1 (3 participants) to day 5 (12 participants). This pattern of results is represented by the number of bad effects in the MixTest (Miller, 2006). Our finding aligns with the previous analysis of mean ITs (see Böer et al., 2024), which indicated that participants were able to overcome the fake-production costs at the ISI of 400 ms with increasing practice.

The findings of persistent cognitive costs with no mental preparation time (i.e., ISI 0 ms) and of reduced, or even eliminated, fake-production costs with some mental preparation time (i.e. ISI 400 ms), are of interest for the applied field: The uniform effect at ISI 0 ms means that players always show fake-production costs when performing head fakes without mentally preparing the action beforehand. This could be a disadvantage for the attacking player, as research suggests that an increase in the ITs of a fake action increases the chance that the opponent expects a deceptive movement (Kunde et al., 2019). Conversely, the mixed effect at the ISI 400 ms indicates that participants only show fake-production costs in some trials, but in those cases the fake-production costs are significantly higher. The attacking player therefore only has a disadvantage in those few trials, in which ITs are substantially slowed when performing a pass with head fake, as the opponent would have a better chance to correctly anticipate the fake action (cf. Kunde et al., 2019). However, in the trials where ITs are not slowed, this disadvantage vanishes. Overall, the analysis confirms our previous conclusion in Böer et al. (2024), that it is helpful to mentally prepare the execution of a deceptive action shortly beforehand. Furthermore, practicing fake movements and reducing the time needed to mentally prepare the movement is essential to gain an advantage over the defending player.

### Comparison of mean ITs between the practice and the control group

The results of this analysis revealed that the practice group exhibited significantly greater reductions in ITs for both passes with and without head fakes compared to the control group, who did not improve at all. This finding suggests that the improvements observed in the practice group are not merely simple test–retest effects but reflect genuine performance enhancements due to practice with the task.

Interestingly, the current analysis did not indicate a specific reduction of fake-production costs with practice as the four-way interaction of the between-subjects factor *group* with the within-subjects factors *ISI*, *type of pass,* and *day* did not reach significance. This finding contrasts with the results of the practice group reported in Böer et al. (2024). However, the ANOVA indicated a significant interaction between the three within-subjects factors, with post-hoc tests revealing significant reductions of fake-production costs at the ISI 0 ms as well as at the ISI 400 ms. The reduction of fake-production costs appears to be mediated primarily by the performance improvements of the practice group, as the control group did not show any significant improvements from day 1 to day 5.

This result was surprising as one day of practice resulted in great improvements of participants' ITs in the practice group on the following day (day 1 to day 2; cf. Böer et al., 2024), which assumes that also the control group should show this performance improvement. As this is not the case, this indicates that these effects of only one day of practice may instead be short-lived, as the control group did not show any significant improvements on day 5 compared to day 1 (i.e., potential effects of practice on day 1 did not persist to day 5).

The significant main effect of the factor *type of pass*, with slower ITs for passes with head fakes, underscores the increased cognitive load and decision-making complexity associated with performing deceptive actions (Güldenpenning et al., 2023; Kunde et al., 2019; Wood et al., 2017). Similarly, the main effect of *ISI*, showing reduced ITs with increasing ISI length, supports the notion that longer mental preparation times facilitate better performance. This finding is consistent with theories of motor preparation in optimizing response execution (Welford, 1980).

### Implications of the distribution analyses

The analyses of the ex-Gaussian distribution parameters (mu, sigma, tau) offer a sophisticated way of analysing IT data by decomposing response latencies into distinct components. It should be noted, however, that the interpretation of these parameters is debated in the literature (Matzke & Wagenmakers, 2009; Osmon et al., 2018). When used cautiously, it can be a helpful tool to describe IT data (Fitousi, 2020) and uncover differences between experimental conditions that weren’t visible in the analysis of mean values (Ratcliff, 1979).

Mu, the distribution mean, reflects the general response speed and is suggested to encapsulate motor processes (i.e., response planning, response selection; Luce, 1986). Sigma, the distribution variance, reflects the consistency of participants' responses, which has been linked to attentional control (Schmiedek et al., 2007). Tau describes the skew of the distribution, indicating the presence of long ITs in some trials or attentional lapses, which are associated with high task complexity and increased cognitive demands, e.g., in working memory (Engle et al., 1999; Heathcote et al., 1991; Logan, 1988; Tse et al., 2010). Together, these parameters provide a multidimensional view of performance, offering insights that might remain hidden in simple mean IT analyses, particularly in tasks involving varying levels of cognitive and motor demands.

Mu was significantly higher for passes with head fakes than for those without head fakes at the ISI of 0 ms, confirming its role as a marker of motor programming costs (Heuer, 1995). The lack of differences for passes with head fakes and passes without head fakes at ISI 400 ms suggests that even brief mental preparation time can mitigate these programming demands for response-response incompatible movements. As the analysis of mu showed, there were no practice improvements beyond test–retest effects, which indicates that motor planning processes may require prolonged or domain-specific practice to improve significantly. Future studies might explore whether mu values are lower among basketball experts, who likely refine motor programming over years of deliberate practice (Ericsson et al., 1993).

While sigma decreased from day 1 to day 5 for both the practice and control group, these reductions did not differ significantly between groups, supporting the conclusion that they reflect general test–retest effects. This suggests that even brief exposure to the task enables participants to stabilize their performance by reducing response variability, a finding consistent with broader RT research (Heathcote et al., 1991).

Tau consistently reflected the increased cognitive demands of performing passes with compared to passes without head fakes. Across all conditions, tau was higher for passes with head fakes, supporting the hypothesis that it captures the attentional costs arising from resolving the response-response incompatibility. This indicates that response-response incompatibility elongates the distribution’s tail by increasing attentional lapses in certain trials. Tau seems to be a good general parameter for the increased complexity of the response-response incompatibility associated with performing passes with head fakes, reflecting the increased cognitive demands even at the longer ISIs of 800 ms and 1200 ms which were not evident in simple mean analyses (cf. Böer et al., 2024). Notably, reductions in tau from day 1 to day 5 were only significant in the practice group, suggesting that practice decreases attentional lapses by increasing task automatization. This aligns with the notion that repeated practice leads to performance transitions from algorithmic processing to fast memory retrieval, resulting in reduced cognitive load and improved task performance (Logan, 1988).

The distribution analyses highlighted potentially distinct cognitive processes as the source of fake-production costs at different ISIs. At the ISI 0 ms, mu was the primary source of fake-production costs, which we take as a strong indicator that mu reflects higher motor programming costs, connected to planning response-response incompatible movements (cf. Heuer, 1995). We therefore argue that mu encapsulates motor processes (i.e., response planning, response selection) as also suggested by Luce (1986). In contrast, at the ISI 400 ms, tau became the dominant factor, indicating that some trials remained cognitively demanding despite reduced programming costs. These findings suggest that brief mental preparation allows participants to address motor demands effectively but does not eliminate attentional lapses, which persist in some trials and elongate the distribution’s tail (Schmiedek et al., 2007). The parameter sigma seemingly did not reflect any distinctions in the cognitive processes associated with producing passes with head fakes or without head fakes, but was merely modulated by test–retest effects, indicating reduced response variability.

### Further theoretical implications

These results, together with the analysis of mixture effects for the ISI 0 ms and 400 ms in the practice group, indicate that there might be distinct cognitive processes involved when performing a pass with head fake without (ISI 0 ms) vs. with mental preparation time (ISI 400 ms). The uniform effect observed at ISI 0 ms indicates consistent fake-production costs affecting most trials, which is reflected in significantly higher values of the distribution mean (mu) for passes with head fakes. This pattern at ISI 0 ms persisted throughout the practice sessions. Conversely, at ISI 400 ms we found a mixed effect with participants showing fake-production costs only in some, but not all trials. This is reflected in the distribution parameters, as we only found differences between passes with head fakes and passes without head fakes in the distributions skew (tau) but not in mu.

If the exact same cognitive process were involved, an increase in preparation time should not change the shape of the distribution, but only shift the distribution to the left. In that case we only should have found a decrease in the mu with increasing ISI, as the processes could be started and finished before participants had to initiate the movement (in line with the widely accepted notion that specific cognitive processes generally take the same amount of time in an individual; cf. Sternberg, 1969). In contrast, the tail of the distribution (tau) should not have differed between different ISIs when the same cognitive processes would have taken place just at different points of time (as long as no real data were removed by the outlier filtering at the short ISIs). But our analyses indicated that the sources of fake-production costs differ between the two ISIs (i.e., mu at ISI 0 ms & tau at ISI 400 ms) as also shown by the different patterns found in the analysis of mixture effects and in their modulation by practice. These results emphasize the necessity of analysing the full IT distribution for a more detailed picture of the effects of different task demands.

### Limitations

The first limitation specifically refers to the distributional analyses conducted here. The number of trials for each condition (*ISI*, *type of pass,* *day*) for each participant (n = 40) was rather small compared to the best-case recommendations for distribution analysis methods detailed in previous research. Increasing the number of trials decreases the chance that the estimated distribution deviates from the actual underlying IT distribution (Lacouture & Cousineau, 2008). However, it should be noted though that the ideal number of trials might not be necessary in this case, as research has shown that for distinct RT distributions like the ex-Gaussian distribution good fitting distributions can be found with as little as 20 reaction times per participant and condition (Ratcliff, 1979).

Further limitations refer to the experimental setup, which has also been discussed in detail in the previous paper (Böer et al., 2024): Participants did not have to decide on their own when and how to play the pass. Instead, they just had to react as fast as possible to a fixed auditory cue. While this oversimplification of a real situation in basketball enabled us to investigate the characteristics of fake-production costs caused by response-response incompatibility, it limits the transferability of our findings to basketball practice. Specifically, it seems that fixed reactions to a specific cue (i.e., always playing a pass to the left side if the right side is blocked by defending player) are easier to execute, than reactions in situations in which the participant could decide for themselves which action to perform (cf. Weller et al., 2018). To improve our understanding of these additional cognitive costs, future research should examine the fake-production costs in a setting where the attacking player him/herself must decide under time constraints which action he/she wants to perform.

Therefore, participants might elicit higher fake-production costs (i.e., longer initiation times) in real game situations in which players must decide under time pressure whether to play a pass with or without a head fake. Also, participants did not have to deceive an opponent, which, however, has been shown to increase cognitive costs potentially as the learnt social rule not to cheat has to be violated (Foerster et al., 2017). Of course, the absence of an opponent in our study limits the transferability of this knowledge to the basketball court. To address this limitation, we started data collection in a social interaction setting where two participants interact with each other, one acting as a defending and the other as an attacking player. This future research hopefully provides more insight into differences of head fake-production costs and might enable us to provide specific recommendations for basketball players and their training routines.

## Conclusion

This study expanded our understanding of fake-production costs in basketball and their modulation through practice. While previous research showed that practicing passes with and without head fakes could reduce fake-production costs, it was unclear whether these improvements were practice-related or simply test–retest effects (Böer et al., 2024). To address this, we analyzed mixture effects (Miller, 2006) in the practice group, and data of a control group was collected for comparisons of mean ITs and distribution parameters.

Our results revealed key differences in how mental preparation time affects the modulation of fake-production costs through practice. Without time to prepare (ISI 0 ms), participants consistently exhibited fake-production costs, even after four days of practice (uniform effect). In contrast, a short preparation time (ISI 400 ms) allowed participants to avoid fake-production costs in some trials (mixed effect), with half of the practice group overcoming these costs entirely by day 5. This suggests that different cognitive processes underlie fake-production costs, depending on whether the action is mentally prepared beforehand.

Practice generally improved ITs across all conditions, with no specific reductions in fake-production costs beyond test–retest effects. Distribution analyses indicated these improvements were largely due to increased task automatization, as reflected by reduced attentional lapses (tau).

Together, these findings emphasize the value of incorporating time-constrained exercises in basketball training to improve performance in deceptive actions. Basketball players should consider mentally preparing for head fakes, as this can generally reduce initiation times and specifically lower fake-production costs, making deceptive actions harder to predict (Kunde et al., 2019). While our study faced limitations, such as an oversimplified task and the absence of opponent deception, future research should explore more realistic scenarios to better understand cognitive costs in real games. In essence, our findings expand the understanding of head-fake production in basketball and provide evidence that analysing the full distribution of ITs offers valuable insight into the cognitive processes underlying deceptive actions.

## Data Availability

Data is provided under the Open Science Framework following this link: https://osf.io/svjtz/

## Footnotes

1 In the study of Böer et al. (2024) participants had 5 days of practice. Figure 5 in the section results part II; Comparison of the mean ITs between the practice and the control group, only displays day 1 and day 5 because the control group was only tested on these two days.

## References

[CR1] Böer, N. T., Weigelt, M., Schütz, C., & Güldenpenning, I. (2024). Practice reduces the costs of producing head fakes in basketball. *Psychological Research, 88,* 523–534. 10.1007/s00426-023-01885-x 37831215 10.1007/s00426-023-01885-x PMC10858151

[CR2] Compton, B. J., & Logan, G. D. (1991). The transition from algorithm to retrieval in memory-based theories of automaticity. *Memory & Cognition,**19*(2), 151–158. 10.3758/BF03197111 2017038 10.3758/bf03197111

[CR3] Dayan, E., & Cohen, L. G. (2011). Neuroplasticity subserving motor skill learning. *Neuron,**72*(3), 443–454. 10.1016/j.neuron.2011.10.008 22078504 10.1016/j.neuron.2011.10.008 PMC3217208

[CR4] Engle, R. W., Tuholski, S. W., Laughlin, J. E., & Conway, A. R. (1999). Working memory, short-term memory, and general fluid intelligence: A latent-variable approach. *Journal of Experimental Psychology: General,**128*(3), 309–331. 10.1037/0096-3445.128.3.309 10513398 10.1037//0096-3445.128.3.309

[CR5] Ericsson, K. A., Krampe, R. T., & Tesch-Römer, C. (1993). The role of deliberate practice in the acquisition of expert performance. *Psychological Review,**100*, 363–406. 10.1037/0033-295X.100.3.363

[CR6] Fitousi, D. (2020). Linking the ex-gaussian parameters to cognitive stages: Insights from the linear ballistic accumulator (lba) model. *The Quantitative Methods for Psychology,**16*, 91–106. 10.20982/tqmp.16.2.p091

[CR7] Foerster, A., Wirth, R., Herbort, O., Kunde, W., & Pfister, R. (2017). Lying upside-down: Alibis reverse cognitive burdens of dishonesty. *Journal of Experimental Psychology: Applied,**23*(3), 301–319. 10.1037/xap0000129 28557488 10.1037/xap0000129

[CR8] Friehs, M. A., Güldenpenning, I., Frings, C., & Weigelt, M. (2019). Electrify your game! Anodal tDCS increases the resistance to head fakes in basketball. *Journal of Cognitive Enhancement, 4, *62–70. 10.1007/s41465-019-00133-8

[CR9] Güldenpenning, I., Alhaj Ahmad Alaboud, M., Kunde, W., & Weigelt, M. (2018). The impact of global and local context information on the processing of deceptive actions in game sports. *German Journal of Exercise and Sport Research,**48*(3), 366–375. 10.1007/s12662-018-0493-4

[CR11] Güldenpenning, I., Kunde, W., & Weigelt, M. (2017). How to trick your opponent: A review article on deceptive actions in interactive sports. *Frontiers in Psychology,**8*, 917. 10.3389/fpsyg.2017.00917 28620336 10.3389/fpsyg.2017.00917 PMC5449506

[CR12] Güldenpenning, I., Kunde, W., & Weigelt, M. (2020a). Cognitive load reduces interference by head fakes in basketball. *Acta Psychologica,**203*, 103013. 10.1016/j.actpsy.2020.103013 31955031 10.1016/j.actpsy.2020.103013

[CR14] Güldenpenning, I., Schütz, C., Weigelt, M., & Kunde, W. (2020b). Is the head-fake effect in basketball robust against practice? Analyses of trial-by-trial adaptations, frequency distributions, and mixture effects to evaluate effects of practice. *Psychological Research,**84*(3), 823–833. 10.1007/s00426-018-1078-4 30128660 10.1007/s00426-018-1078-4

[CR13] Güldenpenning, I., Kunde, W., & Weigelt, M. (2022). Head-fake perception in basketball: The relative contributions of expertise, visual or motor training, and test repetition. *International Journal of Sport and Exercise Psychology,**20*(1), 202–222. 10.1080/1612197X.2020.1854819

[CR15] Güldenpenning, I., Weiglt, M., Böer, N. T., & Kunde, W. (2023). Producing deceptive actions in sports: The costs of generating head fakes in basketball. *Human Movement Science,**87*, 103045. 10.1016/j.humov.2022.103045 36508851 10.1016/j.humov.2022.103045

[CR10] Güldenpenning, I., Böer, N. T., Kunde, W., Giesen, C. G., Rothermund, K., & Weigelt, M. (2024). Context-specific adaptation for head fakes in basketball: A study on player-specific fake-frequency schedules. *Psychological Research, 88*, 1702–1711. 10.1007/s00426-024-01977-2 38806734 10.1007/s00426-024-01977-2 PMC11281954

[CR16] Hazeltine, E. (2005). Response-response compatibility during bimanual movements: Evidence for the conceptual coding of action. *Psychonomic Bulletin & Review,**12*(4), 682–688. 10.3758/BF03196758 16447382 10.3758/bf03196758

[CR17] Hazeltine, E., Diedrichsen, J., Kennerley, S. W., & Ivry, R. B. (2003). Bimanual cross-talk during reaching movements is primarily related to response selection, not the specification of motor parameters. *Psychological Research,**67*(1), 56–70. 10.1007/s00426-002-0119-0 12589450 10.1007/s00426-002-0119-0

[CR18] Heathcote, A., Popiel, S. J., & Mewhort, D. J. (1991). Analysis of response time distributions: An example using the Stroop task. *Psychological Bulletin,**109*(2), 340. 10.1037/0033-2909.109.2.340

[CR19] Hervey, A. S., Epstein, J. N., Curry, J. F., Tonev, S., Eugene Arnold, L., Keith Conners, C., & Hechtman, L. (2006). Reaction time distribution analysis of neuropsychological performance in an ADHD sample. *Child Neuropsychology,**12*(2), 125–140. 10.1080/09297040500499081 16754533 10.1080/09297040500499081

[CR20] Heuer, H. (1995). Models for response-response compatibility: The effects of the relation between responses in a choice task. *Acta Psychologica,**90*(1–3), 315–332. 10.1016/0001-6918(95)00023-N

[CR21] Heuer, H., Spijkers, W., Kleinsorge, T., van der Loo, H., & Steglich, C. (1998). The time course of cross-talk during the simultaneous specification of bimanual movement amplitudes. *Experimental Brain Research,**118*, 381–392. 10.1007/s002210050292 9497145 10.1007/s002210050292

[CR22] Hockley, W. E. (1984). Analysis of response time distributions in the study of cognitive processes. *Journal of Experimental Psychology: Learning, Memory, and Cognition,**10*(4), 598. 10.1037/0278-7393.10.4.598

[CR23] Hohle, R. H. (1965). Inferred components of reaction times as functions of foreperiod duration. *Journal of Experimental Psychology,**69*(4), 382. 10.1037/h0021740 14286308 10.1037/h0021740

[CR24] Holm, S. (1979). A simple sequentially rejective multiple test procedure. *Scandinavian Journal of Statistics,**6*(2), 65–70.

[CR25] Hong, J. Y., Gallanter, E., Müller-Oehring, E. M., & Schulte, T. (2019). Phases of procedural learning and memory: Characterisation with perceptual-motor sequence tasks. *Journal of Cognitive Psychology,**31*(5–6), 543–558. 10.1080/20445911.2019.1642897 33868637 10.1080/20445911.2019.1642897 PMC8048153

[CR26] Jackson, R. C., & Cañal-Bruland, R. (2019). Deception in sport. In A. M. Williams & R. C. Jackson (Eds.), *Anticipation and decision making in sport* (pp. 99–116). Routledge.

[CR27] Kane, M. J., & Engle, R. W. (2003). Working-memory capacity and the control of attention: The contributions of goal neglect, response competition, and task set to Stroop interference. *Journal of Experimental Psychology: General,**132*(1), 47. 10.1037/0096-3445.132.1.47 12656297 10.1037/0096-3445.132.1.47

[CR28] Kunde, W., Foerster, A., Weigelt, M., & Dignath, D. (2019). On the ball: Short-term consequences of movement fakes. *Acta Psychologica,**198*, 102872. 10.1016/j.actpsy.2019.102872 31254864 10.1016/j.actpsy.2019.102872

[CR29] Kunde, W., Skirde, S., & Weigelt, M. (2011). Trust my face: Cognitive factors of head fakes in sports. *Journal of Experimental Psychology: Applied,**17*(2), 110–127. 10.1037/a0023756 21604910 10.1037/a0023756

[CR30] Lacouture, Y., & Cousineau, D. (2008). How to use MATLAB to fit the ex-Gaussian and other probability functions to a distribution of response times. *Tutorials in Quantitative Methods for Psychology.,**4*(1), 35–45. 10.20982/tqmp.04.1.p035

[CR31] Logan, G. D. (1988). Toward an instance theory of automatization. *Psychological Review,**95*(4), 492. 10.1037/0033-295X.95.4.492

[CR32] Logan, G. D. (1992). Shapes of reaction-time distributions and shapes of learning curves: A test of the instance theory of automaticity. *Journal of Experimental Psychology: Learning, Memory, and Cognition,**18*(5), 883–914. 10.1037/0278-7393.18.5.883 1402715 10.1037//0278-7393.18.5.883

[CR33] Luce, R. D. (1986). *Response times: Their role in inferring elementary mental organization (No. 8)*. USA: Oxford University Press. 10.1093/acprof:oso/9780195070019.001.0001

[CR34] Matzke, D., & Wagenmakers, E. J. (2009). Psychological interpretation of the ex-Gaussian and shifted Wald parameters: A diffusion model analysis. *Psychonomic Bulletin & Review,**16*, 798–817. 10.3758/PBR.16.5.798 19815782 10.3758/PBR.16.5.798

[CR35] Mewhort, D. J., Braun, J. G., & Heathcote, A. (1992). Response time distributions and the Stroop Task: A test of the Cohen, Dunbar, and McClelland (1990) model. *Journal of Experimental Psychology: Human Perception and Performance,**18*(3), 872. 10.1037/0096-1523.18.3.872 1500881 10.1037//0096-1523.18.3.872

[CR36] Miller, J. (2006). A likelihood ratio test for mixture effects. *Behavior Research Methods,**38*(1), 92–106. 10.3758/BF03192754 16817518 10.3758/bf03192754

[CR37] Osman, A., Lou, L., Muller-Gethmann, H., Rinkenauer, G., Mattes, S., & Ulrich, R. (2000). Mechanisms of speed–accuracy tradeoff: Evidence from covert motor processes. *Biological Psychology,**51*(2–3), 173–199. 10.1016/S0301-0511(99)00045-9 10686365 10.1016/s0301-0511(99)00045-9

[CR38] Osmon, D. C., Kazakov, D., Santos, O., & Kassel, M. T. (2018). Non-Gaussian distributional analyses of reaction times (RT): Improvements that increase efficacy of RT tasks for describing cognitive processes. *Neuropsychology Review,**28*, 359–376. 10.1007/s11065-018-9382-8 30178182 10.1007/s11065-018-9382-8

[CR39] Peterson, J. R. (1965). Response-response compatibility effects in a two-hand pointing task. *Human Factors,**7*(3), 231–236. 10.1177/001872086500700305 5867018 10.1177/001872086500700305

[CR40] Polzien, A., Güldenpenning, I., & Weigelt, M. (2020). Examining the perceptual-cognitive mechanism of deceptive actions in sports. *Experimental Psychology,**67*(6), 349–363. 10.1027/1618-3169/a000503 33661040 10.1027/1618-3169/a000503

[CR41] Polzien, A., Güldenpenning, I., & Weigelt, M. (2021). A question of (perfect) timing: A preceding head turn increases the head-fake effect in basketball. *PLoS ONE,**16*(5), e0251117. 10.1371/journal.pone.0251117 33979374 10.1371/journal.pone.0251117 PMC8115800

[CR42] Ratcliff, R. (1979). Group reaction time distributions and an analysis of distribution statistics. *Psychological Bulletin,**86*(3), 446. 10.1037/0033-2909.86.3.446 451109

[CR43] Richardson, J. T. (2011). Eta squared and partial eta squared as measures of effect size in educational research. *Educational Research Review,**6*(2), 135–147. 10.1016/j.edurev.2010.12.001

[CR44] Schmiedek, F., Oberauer, K., Wilhelm, O., Süß, H. M., & Wittmann, W. W. (2007). Individual differences in components of reaction time distributions and their relations to working memory and intelligence. *Journal of Experimental Psychology: General,**136*(3), 414. 10.1037/0096-3445.136.3.414 17696691 10.1037/0096-3445.136.3.414

[CR45] Sternberg, S. (1969). The discovery of processing stages: Extensions of Donders’ method. *Acta Psychologica,**30*, 276–315. 10.1016/0001-6918(69)90055-9

[CR46] Tse, C. S., Balota, D. A., Yap, M. J., Duchek, J. M., & McCabe, D. P. (2010). Effects of healthy aging and early stage dementia of the Alzheimer’s type on components of response time distributions in three attention tasks. *Neuropsychology,**24*(3), 300. 10.1037/a0018274 20438208 10.1037/a0018274 PMC2864950

[CR47] Vasquez, B. P., Binns, M. A., & Anderson, N. D. (2018). Response time consistency is an indicator of executive control rather than global cognitive ability. *Journal of the International Neuropsychological Society,**24*(5), 456–465. 10.1017/S1355617717001266 29208077 10.1017/S1355617717001266

[CR48] Weigelt, M., Güldenpenning, I., & Steggemann-Weinrich, Y. (2020). The head-fake effect in basketball is based on the processing of head orientation, but not on gaze direction. *Psychology,**11*(10), 1493–1510. 10.4236/psych.2020.1110095

[CR49] Welford, W. T., Brebner, J. M., & Kirby, N. (1980). *Reaction times*. Stanford University.

[CR50] Weller, L., Kunde, W., & Pfister, R. (2018). Disarming the gunslinger effect: Reaction beats intention for cooperative actions. *Psychonomic Bulletin & Review,**25*(2), 761–766. 10.3758/s13423-018-1462-5 29623572 10.3758/s13423-018-1462-5

[CR51] Whelan, R. (2008). Effective analysis of reaction time data. *The Psychological Record,**58*, 475–482. 10.1007/BF03395630

[CR52] Wood, G., Vine, S. J., Parr, J., & Wilson, M. R. (2017). Aiming to deceive: Examining the role of the quiet eye during deceptive aiming actions. *Journal of Sport & Exercise Psychology,**39*(5), 327–338. 10.1123/jsep.2017-0016 29185367 10.1123/jsep.2017-0016

